# Not Just *N*_*e*_  *N*_*e*_-More: New Applications for SMC from Ecology to Phylogenies

**DOI:** 10.1093/gbe/evaf229

**Published:** 2025-11-28

**Authors:** David Peede, Trevor Cousins, Arun Durvasula, Anastasia Ignatieva, Toby G L Kovacs, Alba Nieto, Emily E Puckett, Elizabeth T Chevy

**Affiliations:** Department of Ecology, Evolution, and Organismal Biology, Brown University, Providence, RI 02912, USA; Center for Computational Molecular Biology, Brown University, Providence, RI 02912, USA; Institute at Brown for Environment and Society, Brown University, Providence, RI 02912, USA; Department of Genetics, University of Cambridge, Cambridge CB2 3EH, UK; Division of Epidemiology, Department of Population and Public Health Sciences, Keck School of Medicine, University of Southern California, Los Angeles, CA 90033, USA; Center For Genetic Epidemiology, Keck School of Medicine, University of Southern California, Los Angeles, CA 90033, USA; Department of Quantitative and Computational Biology, University of Southern California, Los Angeles, CA 90089, USA; Department of Statistics, University of Oxford, Oxford OX1 3LB, UK; School of Life and Environmental Sciences, University of Sydney, Sydney, NSW 2006, Australia; Institut de Systématique, Evolution, Biodiversité (ISYEB), Muséum national d’Histoire naturelle, CNRS, Sorbonne Université, Université des Antilles, 75005 Paris, France; École Pratique des Hautes Études (EPHE), PSL Research University, Paris 75006, France; Department of Biological Sciences, University of Memphis, Memphis, TN 38111, USA; Department of Ecology, Evolution, and Organismal Biology, Brown University, Providence, RI 02912, USA; Center for Computational Molecular Biology, Brown University, Providence, RI 02912, USA

**Keywords:** sequentially Markovian coalescent, demographic inference, gene flow, conservation biology, ARG reconstruction

## Abstract

Genomes contain the mutational footprint of an organism’s evolutionary history, shaped by diverse forces including ecological factors, selective pressures, and life history traits. The sequentially Markovian coalescent (SMC) is a versatile and tractable model for the genetic genealogy of a sample of genomes, which captures this shared history. Methods that utilize the SMC, such as PSMC and MSMC, have been widely used in evolution and ecology to infer demographic histories. However, these methods ignore common biological features, such as gene flow events and structural variation. Recently, there have been several advancements that widen the applicability of SMC-based methods: inclusion of an isolation with migration model, integration with the multi-species coalescent, incorporation of ecological life history traits (such as selfing and dormancy), and many computational advances in applying these models to data. We give an overview of the SMC model and its various recent extensions, discuss examples of biological discoveries through SMC-based inference, and comment on the assumptions, benefits and drawbacks of various methods.

Significance Inferring historical population size ($N_{e}$) from genome sequences is common across fields such as population genetics, natural history, and ecology. The methods that generate these inferences (e.g., PSMC, MSMC) share a common statistical framework, the sequentially Markovian coalescent. Recent extensions to this framework now allow for the inference of quantities and forces beyond $N_{e}$, such as population structure, gene flow, or ecologically relevant life history traits, widening its applicability to larger and more varied datasets. In an effort to extend awareness of these new possibilities across fields, we review how these extensions work, where they have been applied, and key empirical considerations that influence their effective use.

## Introduction

Detecting signals of historic demographic events, such as population size changes, given DNA samples from a population is a fundamental goal of population geneticists, natural historians, and conservation biologists alike. The coalescent (Kingman 1980, 1982a, 1982b) and its subsequent extensions that account for recombination (Hudson 1983; Griffiths 1991) provided the probabilistic foundations for the development of statistical inference methods that estimate demographic parameters from inferred sample genealogies—for a comprehensive overview, see Wakeley (2008). These coalescent models trace ancestral relationships backwards in time for a set of sampled lineages, where a coalescence event occurs when two lineages merge into a single ancestral lineage at some point in the past. A key parameter of interest is the effective population size ($N_{e}$), which can be conceptualized as the size of an idealized Wright-Fisher population that matches the coalescence rate of the population under study (Fisher 1923; Wright 1931; Charlesworth 2009). An alternative yet convenient perspective is to define $N_{e}$ as the inverse of the coalescence rate, as this definition can flexibly encapsulate various properties of an evolutionary model.

For nonrecombining segments of DNA—such as mitochondrial DNA (mtDNA) and chloroplast DNA (cpDNA)—the genealogical history of a sample can be represented by a single tree structure. However, a recombination event will split a segment of DNA into two, resulting in distinct genealogical histories for the two recombinant segments. The coalescent with recombination (CwR) was originally formulated as a backwards in time process that models the ancestral relationships for a set of samples while accounting for recombination events (Hudson 1983). The CwR can be conceptualized in two distinct ways: as a collection of local trees or as a graph structure known as the ancestral recombination graph (ARG; Griffiths 1991; Griffiths and Marjoram 1996, 1997). The ARG can encode both coalescence events (where lineages merge) and recombination events (where lineages split), thereby describing the complete evolutionary history for a set of samples at every genomic position. In contrast to its initial formulation, the CwR can alternatively be viewed as a sequential process along the genome rather than backwards in time (Wiuf and Hein 1999a, 1999b). In this perspective, a local genealogy persists along the genome until a recombination event induces a change in tree structure. Inferring the latent ARG of a set of sequences is notoriously difficult, due to the observed data (i.e., the distribution of mutations) not being not very informative about the underlying genealogical history, combined with the fact that the space of possible genealogical histories is extremely large.

To overcome the computational challenges of inference under the CwR, McVean and Cardin (2005) proposed the sequentially Markovian coalescent (SMC) as a tractable approximation to this model, which was further refined by Marjoram and Wall (2006). The SMC framework ushered in a new suite of methods based on coalescent Hidden Markov Models (HMMs; Hobolth et al. 2007; Li and Durbin 2011; Schiffels and Durbin 2014) designed to infer demographic parameters from genomic data. Notably, Li and Durbin’s PSMC method leveraged the SMC in the transition matrix of its HMM to infer the $N_{e}$ trajectory of humans over time, using a diploid sequence of just one individual. Although the SMC' framework follows the assumptions of the standard coalescent, recent theoretical work has extended the SMC to account for factors such as migration (Wang et al. 2020), structural variation (Ignatieva et al. 2025), and selfing (Strütt et al. 2023). These extensions modify the SMC’s probabilistic model or associated HMM approach to infer quantities beyond $N_{e}$.

This review presents these recent extensions to the SMC that broaden its relevance to fields such as natural history and conservation biology. After a brief primer describing the fundamentals of the SMC framework, we divide its extensions into three broad classes: those doing *joint parameter inference*, those doing *lineage partitioning*, and those building upon the SMC to explicitly infer sample genealogies (in the form of ARGs). For each class, we describe how recent methods extend the SMC, then review examples of their application to empirical (or simulated) data. Finally, as plausible uses of the SMC expand across fields we discuss how empiricists can be best served by these new approaches, and where we expect the SMC framework will continue to prove particularly useful.

## SMC Primer

We begin by briefly defining the SMC model, describing how the SMC is used for inference in the PSMC framework, and reviewing subsequent methodological advances for inferring historical effective population sizes and coalescence rates using SMC-based approaches.

### Coalescent Theory Primer

Kingman’s coalescent (Kingman 1980, 1982a, 1982b) is a probabilistic model that describes the ancestral relationships at a single nonrecombining locus for a set of lineages in an idealized Wright-Fisher population (i.e., panmictic with constant size, nonoverlapping generations, and no selection) of *N* diploid individuals. It arises as the continuous-time approximation to the discrete-generation Wright-Fisher model when the limit $N_{e}\to \infty$, providing a tractable framework for modeling ancestral relationships when *N* is large. In this limit, rescaling time (*t*) leads to a continuous-time process, where time is measured in coalescent units going backwards in time (i.e., t=0 is the present). This can be converted back to time measured in generations through multiplying by a factor of $2N_{e}$, the expected time for a pair of lineages to coalesce. Note that, for real populations, the effective population size $N_{e}$ is in general not equal to the census size of a population, reflecting the fact that the accumulation of genetic diversity is affected by violations of the neutral Wright-Fisher model. Note also that, while the Wright-Fisher model is one of the simplest examples for which sample genealogies converge to Kingman’s coalescent, this is also generally the case for a broad class of finite population models (Möhle 2000).

The relationship among sequences sampled from this population can be described with a genealogical tree. It traces the ancestry for a set of lineages backwards in time, and when a pair of lineages share a common ancestor they are said to *coalesce*, which is represented through the two lineages merging in the tree. The coalescent characterizes the distribution over the shapes of such trees, and the waiting times between coalescence events. When considering a sample of size two, there is only one possible tree topology, so the coalescent fully characterizes the sample genealogy through the distribution of the time of their most recent common ancestor.

### The Coalescent with Recombination (CwR)

While Kingman’s coalescent can be effectively applied independently to unlinked loci, nearby loci have highly correlated genealogies, so the effects of recombination must be modeled explicitly. The CwR was first formulated by Hudson (1983) as a stochastic process backwards in time, which allows for both coalescence and recombination events to occur, resulting in a collection of local trees. Further developments introduced an alternative graph structure to represent a realization of this stochastic process, where lineages can both merge due to coalescence and split due to recombination (Griffiths 1991; Griffiths and Marjoram 1996, 1997). This graph structure, known as the ARG, encodes the complete evolutionary history of a sample, including the marginal genealogy at each genomic position (Griffiths 1991; Griffiths and Marjoram 1996, 1997). Rather than representing the CwR as a stochastic process backwards in time, Wiuf and Hein (1999a) reframed it as a spatial process that sequentially generates correlated local genealogies along the chromosome. Under this spatial formulation, the local genealogy at any given position depends on all preceding genealogies, making the process non-Markovian. This introduces long-range dependencies between genomic regions where nonancestral segments are “trapped” by flanking ancestral segments, rendering the spatial formulation challenging for tractable inference.

The ARG has been described as “*the holy grail of statistical population genetics*” because it can fully encode the entire genealogical history of the sample, including the complete record of which lineages experienced recombination and the time when these events occurred (Hubisz and Siepel 2020). Given a set of sampled genomes, it would be desirable to infer an ARG that could recover the topologies of local genealogies, the timing of coalescence events, the ages of mutations, and the locations and times of recombination events. However, the complexity and size of the space of possible ARGs make population genetic inference under the CwR extraordinarily challenging (Griffiths and Marjoram 1996; McVean and Cardin 2005). Even when considering a single locus, the number of possible coalescence topologies for *n* sequences is ∏i=2n(i2)=  $n!(n-1)!/2^{n-1}$ (Hein et al. 2004, Section 3.2), which grows super exponentially, making likelihood calculations for an ARG infeasible for realistic sample sizes and sequence lengths. Indeed, algorithms that compute likelihoods without relying on approximations to the CwR exist for very small datasets (e.g., ARGinfer; Mahmoudi et al. 2022); nevertheless, for most practical applications, simplifying the structure of the ARG and approximating the full CwR remains necessary for tractable *and* computationally scalable inference.

### SMC as an Approximation to the CwR

McVean and Cardin (2005) introduced the SMC as a model to sequentially generate genealogies along a chromosome. In this sequential view, given a recombination event, the SMC permits the “floating” lineage—i.e., the branch below the recombination event—to re-coalesce with any older lineage on the tree except for itself. Like the CwR, the SMC can also be viewed from a backwards in time perspective, where lineages can only coalesce if they share overlapping ancestral material. The SMC is thus a Markov process both backwards in time and along the genome, since the genealogy at any given genomic position depends only on the genealogy at the previous position. Despite this simplification, the SMC generates genealogies with very similar structure, correlation, and patterns of genetic diversity as the CwR, while being much more computationally tractable for inference (McVean and Cardin 2005).

**Fig. 1.**
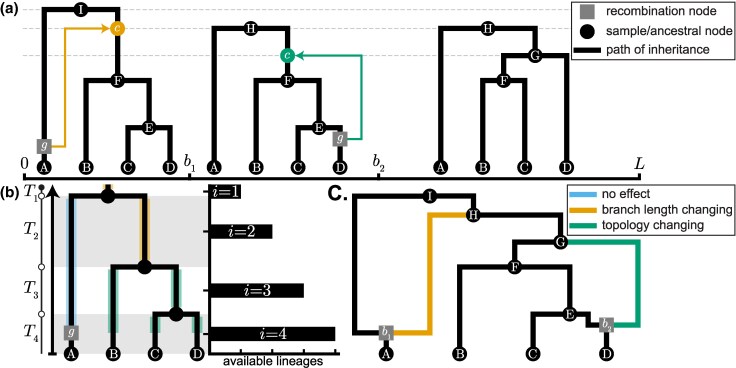
Illustration of the SMC. a) SMC process as a sequence of genealogical trees over a genomic region of length *L*. Each local tree persists until a recombination event (grey square node *g*) occurs at a breakpoint $b_{i}$. The branch between the recombination node and the “floating lineage’s” parent node is then pruned. The genealogy transitions to the next tree along the sequence when the floating lineage is regrafted (colored arrow) via a coalescence event (circle *c* node) colored by the types of recombination events described in b. Dashed horizontal lines at the height of ancestral nodes I, H, and G, highlight that the new genealogies are formed due to the coalescence at the preceding locus. b) Possible types of genealogical transitions conditioned on the recombination event (grey square node *g*). The floating lineage coalesces with one of its ancestral lineages to form the next genealogy along the sequence (see a and c). If the floating lineage coalesces with the lineage highlighted in sky blue, the next tree remains unchanged. Coalescence with the lineage highlighted in orange alters the height of the next tree, whereas coalescence with one of the lineages highlighted in green changes the next tree’s topology. Where the floating lineage coalesces depends on the length of each epoch ($T_{i}$, delineated by alternating backgrounds) and the number of ancestral lineages *i*. The histogram shows the number of lineages *i* available for coalescence in each epoch $T_{i}$, starting from the time of the recombination event *g*; the coalescence rate in epoch $T_{i}$ is $i/2N_{e}$. c) Illustration of an ARG for the corresponding genealogical trees depicted in a. Black circle nodes A-D represent sample nodes, and the remaining ancestral black nodes are alphabetically labeled in ascending order based on relative age. Grey square nodes denote recombination events and are labeled by their corresponding breakpoint $b_{i}$ in a. The left and right edges emerging from recombination nodes correspond to the local genealogies to the left and right of breakpoint $b_{i}$. The orange edge represents the recombination event in the first tree, which shortens the height of the second tree. The green edge represents the recombination event in the second tree, which alters the topology of the final tree.

Marjoram and Wall (2006) introduced a modification to the SMC, known as the SMC' (Fig. 1), which incorporates an additional class of coalescence events, making it a closer approximation to the CwR while still being Markovian. Specifically, in the sequential view, the SMC' also allows the “floating” lineage to re-coalesce with itself, which is not permitted in the original SMC. From the backwards in time perspective, the SMC' allows coalescence between lineages with overlapping *or* adjacent ancestral material, while the SMC only permits coalescence between lineages with overlapping ancestral material. This difference means that after a recombination event, the SMC always generates a new marginal genealogy distinct from the previous one, whereas the SMC' allows for self-coalescence that may preserve the previous genealogical structure in the new marginal genealogy (Marjoram and Wall 2006).

The key insight of both the SMC and the SMC' models is that by making each local genealogy dependent only on the genealogy at the previous locus, the process becomes Markovian. This Markovian approximation substantially reduces the space of possible ARGs, providing a much more tractable framework for statistical inference. Moreover, by studying the joint distribution of coalescence times in a two-locus Markov chain model, Wilton et al. (2015) and Hobolth and Jensen (2014) demonstrated that both the SMC and SMC' are good approximations to the full ARG, with the SMC' being notably more accurate. Furthermore, they showed that the joint distribution of pairwise coalescence times at sites flanking recombination breakpoints under the SMC' is identical to that under the full ARG (Wilton et al. 2015). For these reasons, the SMC' has become the more popular choice for inference methods, and throughout this review, when we refer to “the SMC” and “SMC-based methods” we are, more formally, discussing the SMC'. We describe the SMC' algorithm in Algorithm 1; we note that reversing the order of steps 4 and 5 yields the original SMC formulation described in McVean and Cardin (2005).

**Algorithm 1:** Generating genealogies under the SMC' (Marjoram and Wall 2006).

**Input:** Effective population size $N_{e}$, sequence length *L*, and per-site per-generation recombination rate *r*. Compute the population-scaled recombination rate $\rho =4N_{e}r$.
**1.** Set the first left interval position as x=0 and generate a coalescent tree for *x* under Kingman’s coalescent, denoted as T(x). Denote the length of the tree at *x* as L(x), which sums all of the branch lengths in the tree.
**2.** Generate the right interval position as $y∼\text{Exp}(\frac{\rho }{2}L(x))$, the distance along the chromosome until the next recombination event—i.e., tree T(x) spans the genomic interval [x,x+y).
**3.** Pick a point *g* on the tree T(x) uniformly.
**4.** Add a recombination event to the tree T(x) at point *g*, resulting in a graph, which occurs at chromosomal location x+y. The left emerging branch follows the original path in T(x), while the right emerging branch re-coalesces with a lineage in T(x) at some point *c* higher up on the graph (after a waiting time which is exponentially distributed, with rate proportional to the number of ancestral lineages at each epoch).
**5.** Delete the part of the left emerging branch that lies between the newly added recombination event at point *g* and the next coalescence event along the branch, thus reverting the graph back to a tree.
**6.** Set the new left interval position as $\hat{x}=x+y$, where $T(\hat{x})$ and $L(\hat{x})$ denote the new tree constructed starting from position $\hat{x}$ and its associated tree length.
**7. if** *$\hat{x}<L$* **then**
Set $x=\hat{x}$ and return to step 2.

### Statistical Inference Using the SMC

The most common application of the SMC is to estimate changes in $N_{e}$ over time from sequencing data. To infer model parameters under the SMC conditional on the data, methods such as PSMC or MSMC (and many others reviewed in Sections 2 and 3; Li and Durbin 2011; Schiffels and Durbin 2014) use a Hidden Markov Model (HMM). HMMs are a powerful and flexible inferential framework; indeed, one of the most useful features of the SMC approximation to the CwR is that it is Markovian and therefore allows the use of HMMs—for more about HMMs in this context, see Durbin et al. (1998).

**Fig. 2.**
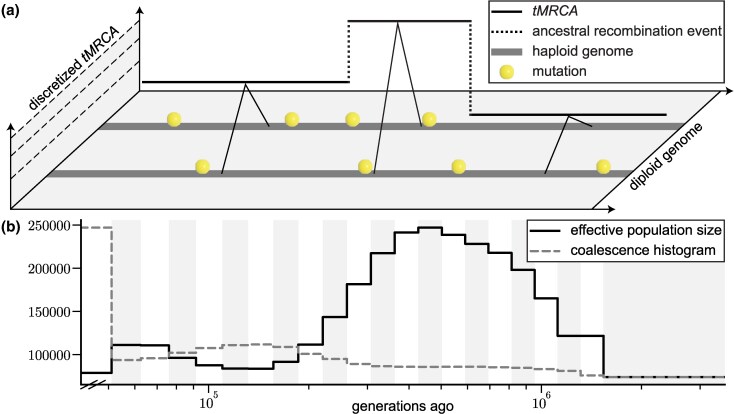
Overview of PSMC framework. a) PSMC is a Hidden Markov Model (HMM) used to infer piecewise constant historical population sizes from the spatial distribution of heterozygous sites between two haploid sequences (grey lines on the *x*-axis plane). Hidden states are discrete intervals of times to the most recent common ancestor (*tMRCA*), shown as dashed lines on the *y*-axis plane. Transitions between hidden states represent changes in the *tMRCA* of the two sequences, reflective of ancestral recombination events (dotted vertical lines). Observations consist of the sequence of diploid genotypes at each site, where a yellow sphere indicates a heterozygous site (i.e., the mutation occurred after the *tMRCA*) and its absence indicates a homozygous site. Emissions correspond to the probability of observing these mutations given a *tMRCA* for the two sequences. b) Using the Baum-Welch algorithm to estimate model parameters, PSMC reconstructs a piecewise constant ancestral population size history. The crux of PSMC’s inference relies on the inverse relationship between the frequency of coalescence events (grey dashed line) in each time interval (alternating background) and the effective population size (black line) during that time period.

PSMC models the density of heterozygous sites along the genome to infer the coalescence time at every genomic position using sequence data from a single diploid individual (Fig. 2). Since only two sequences are analyzed at a time, each genealogical tree along the sequence contains only two lineages and one coalescence time—i.e., the time to the most recent common ancestor (*tMRCA*) for a diploid individual’s two homologous chromosomes. Time—the axis along which lineages in each tree coalesce backwards in time from the present—is partitioned into discrete time intervals to form the *hidden state space* of a discrete HMM. The *observations* in the data are whether or not alleles match (i.e., the genotype is homozygous or heterozygous) at each site in the sequence, where a heterozygous site indicates that a mutation arose more recently than the *tMRCA* of the individual’s two haploid lineages. The *emission probabilities*, based on the infinite sites mutation model, describe the probability that the observation is homozygous or heterozygous, given that the lineages coalesced in a particular time interval, and are determined by the population-scaled mutation rate $\theta =4N_{e}\mu$. The *transition probabilities*, derived from the SMC framework, describe the probability that a locus whose lineages coalesced in one particular time interval is adjacent to a locus whose lineages coalesced in the same or different time interval, and are governed by the population-scaled recombination rate $\rho =4N_{e}r$ and the given evolutionary model.

PSMC uses the Baum-Welch algorithm—a special case of the expectation-maximization algorithm—to estimate its parameters, namely the piecewise constant coalescence rates and the population-scaled recombination rate *ρ*. In the expectation step, the forward-backward algorithm computes the posterior probability of coalescence in each hidden state for each observation, this posterior distribution is then used to construct the expected transition matrix. In the maximization step, the expected transition matrix is used to update the model parameters. The updated parameters are subsequently used to recompute the expected transition matrix, and the process iterates until convergence. The associated time complexity scales linearly with the number of observations (i.e., sequence length) and quadratically with the number of hidden states. After convergence is achieved, $N_{e}$ is estimated from the PSMC’s piecewise constant coalescence rates as the inverse of the coalescence rate, after appropriately scaling the time units using the per-site per-generation mutation rate *μ* and generation time.

#### Inferring Demographic Histories

The ability to infer changes in historical population sizes from a single diploid genome sequence has made PSMC a very commonly used tool in the field of evolutionary genetics (Hilgers et al. 2025). Building on this foundation, several methods have extended PSMC’s capabilities to improve inference of historical population sizes over time. The multiple sequentially Markovian coalescent (MSMC) was introduced as an extension that takes multiple (typically as many as eight) diploid genome sequences as input (Schiffels and Durbin 2014). MSMC estimates the time of the first coalescence event among all input sequences, offering two key advantages over its predecessor: for sequences sampled from the same population, the larger sample size enables greater resolution in population size inference for more recent time periods; for sequences sampled from two different populations, it infers the relative cross coalescence rate—a measure of genetic separation between populations based on the ratio between cross-population and within-population coalescence rates. MSMC also employs a more accurate approximation to the CwR (Marjoram and Wall 2006, i.e., SMC') compared to the original PSMC implementation (McVean and Cardin 2005; Li and Durbin 2011). Building on MSMC, MSMC2 was introduced as a simpler yet more powerful approach (Wang et al. 2020). Rather than inferring the first coalescence event among input sequences, MSMC2 runs PSMC on all possible pairs of sequences and uses composite-maximum likelihood to infer model parameters.

Another class of methods leverages the sequentially Markov conditional sampling distribution (SMCSD), which describes the probability of observing a new haplotype given a set of previously observed haplotypes and a demographic model (Paul and Song 2010; Paul et al. 2011; Steinrücken et al. 2013). diCal employs the SMCSD to infer recent population size history using up to 10 genomes (Sheehan et al. 2013), while diCal2 extends this framework to infer more complex demographic histories involving population splits, admixture, and migration (Steinrücken et al. 2019). SMC_++_ builds on these approaches by combining the SMCSD with explicit modeling of the site frequency spectrum (SFS), enabling analysis of hundreds of unphased genomes (Terhorst et al. 2017). Recently, Terhorst (2025) introduced PHLASH, a Bayesian extension of PSMC and SMC_++_ that infers historical population size changes by placing a prior on the discrete time intervals and sampling from the posterior distribution, thereby removing the need for users to discretize time.

Several other methods have introduced novel approaches to infer population size histories under the SMC framework. SMCSMC uses sequential Monte Carlo to estimate the posterior distribution of the hidden continuous time Markov process from four diploid human genomes, and avoids biases in the expectation-maximization algorithm by employing variational Bayes to model uncertainty in rare events (Henderson et al. 2021). CHIMP scales to hundreds of genomes by modeling the latent space as either the height or length of the tree, obtaining the Markov transition probabilities through numerically solving systems of differential equations, and using composite likelihood to scale to large sample sizes (Upadhya and Steinrücken 2022). Additionally, a Gaussian process-based Bayesian nonparametric method has been developed that avoids discretization of the parameter space while providing uncertainty estimates for inferred parameters (Palacios et al. 2015).

#### Inferring Coalescence Times to Study Natural Selection

While SMC-based methods have primarily focused on inferring demographic histories, the sequence of coalescence times inferred along the genome also provides valuable information for studying natural selection. For example, regions under recent positive selection are enriched for young *tMRCA*s, whereas regions under long-term balancing selection are enriched for ancient *tMRCA*s.

PSMC’s posterior decoding scales quadratically in the number of hidden states, making it impractical to apply pairwise to a large number of sequences. By exploiting symmetries in the discretized SMC transition process, Harris et al. (2014) showed that the PSMC algorithm can be decoded in linear time. The Ascertained Sequentially Markovian Coalescent (ASMC; Palamara et al. 2018) utilizes this linear decoding, combined with efficient dynamic programming, to run pairwise *tMRCA* inference on biobank scale data, finding novel signatures of recent positive selection and illuminating the landscape of selection on complex traits. Further improving the efficiency of PSMC’s posterior decoding (Schweiger and Durbin 2023) introduced Gamma-SMC. Whereas ASMC’s decodes in linear time, Gamma-SMC decodes in constant time. This speedup is accomplished by representing the hidden distribution over the coalescence times with a Gamma distribution, and computing a forward/backward pass through the observed data by using a flow-field to approximate the transition density. Furthermore, the gamma representation allows the *tMRCA*s to be modeled in continuous time and is thus more robust to misspecification.

## SMC with Joint Parameter Inference

**Fig. 3.**
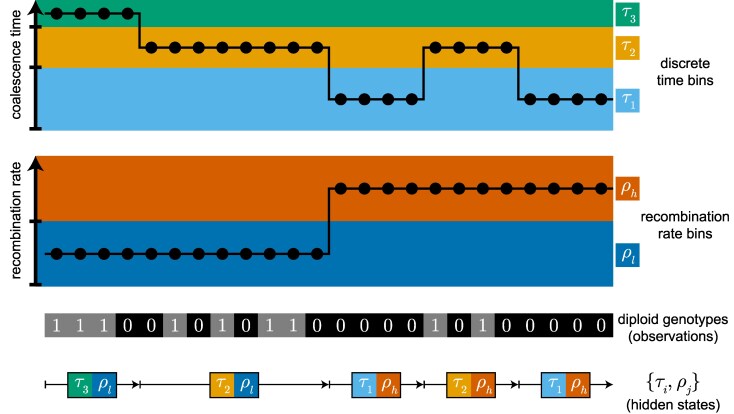
Illustrative example of joint parameter inference. The Hidden Markov Model (HMM) infers both a discretized coalescence time history (vector of $\tau_{i}$) and a discretized recombination rate map (vector of $\rho_{h}$ and $\rho_{l}$, indicating high and low recombination respectively). Hidden states are tuples of all combinations of coalescence time bins $\tau_{i}$ and recombination rate bins $\rho_{j}$. An HMM path is depicted underneath the observed diploid genotypes (“0” for homozygous and “1” for heterozygous), which corresponds to a sequence of hidden states (pairs of $\{\tau_{i},\rho_{j}$}) emitted by the sequence of observations.

Several extensions of the SMC framework can infer not just a vector of inverse coalescence rates (i.e., $N_{e}$) through time, but also vectors of other biological parameters of interest, including mutation and recombination rates, mating system properties, and sequence constraint (Fig. 3).

### SMC-based Inference with Mutation and Recombination Rate Heterogeneity

PSMC brought large inferential power to genome projects by allowing the estimation of historical population sizes from a single unphased diploid genome. The integrative Sequentially Markovian Coalescent (iSMC) extends what can be learned from such data by extending the SMC framework to also estimate a recombination map along the genome (Barroso et al. 2019). In the original PSMC framework, the transition rates between coalescent time bins assumes a constant, global recombination rate parameter. iSMC models spatial variation in recombination rate using a Gamma distribution with *k* discretized categories, allowing for the inference of local recombination rates along the genome.

iSMC is not only able to distinguish between the signals of variable population size and recombination rate in SNP data, but provides more accurate estimates when inferring both concurrently. Although iSMC produces lower resolution recombination maps than linkage disequilibrium (LD) based methods, iSMC is more accurate when only a small number of samples are available, or in the presence of historical population size variation (Barroso et al. 2019; Dutheil 2024). However, iSMC introduces biases if the number of recombination categories *k* is poorly selected (Dutheil 2024). iSMC is also limited to estimating the background recombination rate and cannot detect finer-scale variation such as recombination hotspots. Further development is needed for small genomes and those with high gene conversion rates (Schweizer et al. 2021; Dutheil 2024).

iSMC’s ability to analyze single genomes has enabled the estimation of recombination maps for individuals from diverse human populations, as well as for archaic hominins like Neanderthals and Denisovans (Barroso et al. 2019). These findings show that the recombination landscape divergence aligns closely with the divergence of both extant and extinct lineages. This framework has also enabled the first recombination map estimates for a wide range of nonmodel and threatened species (Robinson et al. 2021; Nouhaud et al. 2022; Cui et al. 2024; da Fonseca et al. 2024; Unneberg et al. 2024). These recombination maps have helped highlight the significance of genome-wide recombination rate variation in local genome evolution. For example, iSMC has been used to show that low-recombining genomic regions in small pelagic European sardines contained increased genetic differentiation between populations, suggesting that local recombination rates can influence population structure (da Fonseca et al. 2024). Low-recombining genomic regions have also been shown to be of particular concern in threatened species, as they are more vulnerable to the effects of linked selection. Directional selection reduces $N_{e}$ at linked sites due to genetic hitchhiking (i.e., Hill-Robertson effect; Charlesworth et al. 1993; Nordborg et al. 1996), reducing local levels of genetic diversity proportionally to the strength of selection and inversely to the local recombination rate (Charlesworth 2009). For regions of the genome with lower recombination rates, this effect is particularly pronounced, where linked selection can create long-lasting reductions in both $N_{e}$ and genetic diversity. Low-recombining regions were more likely to contain runs of homozygosity, a common conservation measure used to indicate inbreeding, in the critically endangered Chinese *Bahaba* fish (Cui et al. 2024). These regions were also more likely to contain high-impact substitutions and highly disruptive variants. Higher recombination rates in smaller chromosomes and distal regions of larger chromosomes helped explain the increased heterozygosity in these regions in the critically endangered California condor (Robinson et al. 2021). However, purging of deleterious alleles, a common advantage to prolonged small $N_{e}$, appears to be more efficient in low-recombining regions in hybrid ant genomes (Nouhaud et al. 2022).

iSMC has been extended to estimate mutation rates across the genome alongside demographic histories and recombination rates (Barroso and Dutheil 2023). This approach helps partition the relative contributions of genetic drift, linked selection, and local mutation rates to the evolution of genetic diversity. For instance, this extension revealed that local mutation rates primarily drive patterns of genetic diversity in *Drosophila melanogaster*, with linked selection playing a secondary role (Barroso and Dutheil 2023). While iSMC models heterogeneity in mutation rate across the genome as additional hidden states using a Gamma distribution with *m* discretized categories in an HMM, other strategies have been developed to overcome PSMC’s assumption of a genome-wide constant mutation rate.

Building on this idea, PSMC+ was introduced by Cousins et al. (2024a) to model the effects of background selection, by extending PSMC’s inference framework to explicitly account for local variation in mutation rates and its impact on estimates of $N_{e}$. A classic model of the effect of background selection on pairwise diversity re-scales the effective population size by a factor *B* to account for loss of diversity due to linkage with alleles experiencing purifying selection (Charlesworth et al. 1993). As mentioned in Section 1.4, PSMC’s emission probabilities are governed by the population-scaled mutation rate, which is assumed to be constant along the genome, and describes the probability of observing a mutation given a coalescence time. PSMC+ modifies PSMC’s emission model to account for local variation in mutation rate by scaling *θ* by a factor of $f_{i}$ for each observation *i*, which reduces bias for inferring $N_{e}$ if the true scaling factor is known. In doing so, PSMC+’s emission probabilities now describe the probability of observing a mutation given a coalescence time *and* local mutation rate scaling factor. The authors show via simulations that this procedure improves the accuracy of estimates of $N_{e}$ through time in the presence of background selection. Using a simple *B*-map based on distance to exons, or simply rescaling *post hoc*, performed similarly to the high-resolution Murphy *B*-map in humans, suggesting that minimal prior knowledge of background selection is required. When not accounting for background selection, the ratio of effective population sizes between X-chromosomal and autosomal regions varies substantially over time, although this could be due to life history or mutation rate variation (Cousins et al. 2024a). It should be noted that while Cousins et al. (2024a) primarily focused on the effect of background selection, PSMC+’s emission model can be used to account for any local variation in mutation rate.

### Detecting Life History Traits with the SMC

SMC-based methods inherit neutral assumptions from Kingman’s coalescent (Section 1.1). However, many systems—especially plants and invertebrates—exhibit ecological life history traits that violate two key assumptions the Wright-Fisher model: sexual reproduction through random mating, and nonoverlapping generations. Recent extensions to the SMC framework have addressed these challenges, yielding effective models for phenomena such as self-fertilization and seed- (or egg-) banking.

Self-fertilization is a nonrandom mating system prevalent in plant and fungal species in which an individual’s gametes are more likely to fuse with its own than those of another individual (Nordborg 2000). Self-fertilization reduces genetic diversity and thus decreases the effective population size, resulting in genealogical trees with shorter branches and fewer mutations and recombination events than those generated under the standard SMC (Nordborg 2000). Seed (or egg) banks are an adaptation to unpredictable environments whereby seeds remain dormant for multiple generations before germinating (Cohen 1966). Seed banks break the assumption of nonoverlapping generations by allowing past generations to contribute genetically to the present. This increases the effective population size, resulting in genealogical trees with longer branches and more mutations and recombination events than those generated under the standard SMC (Lennon et al. 2021).

To explicitly infer these life history traits alongside $N_{e}$, Sellinger et al. (2020) developed the ecological sequentially Markovian coalescent (eSMC). The eSMC can be viewed as a rescaled extension of PSMC: it leverages the fact that self-fertilization and seed banking alter the recombination and mutation processes in opposite directions and distort the ratio of recombination to mutation rates. By exploiting this deviation, the eSMC is able to jointly infer a piecewise constant demographic history along with either a fixed self-fertilization rate or a germination rate.

Applying eSMC to Swedish and German *Arabidopsis thaliana* populations, Sellinger et al. (2020) showed that self-fertilization rates introduce minimal bias into historical $N_{e}$ estimates compared to traditional SMC-based inference methods (Durvasula et al. 2017). Moreover, the eSMC rejected the previously-proposed existence of seed banks in these populations. In contrast, when applied to *Daphnia pulex*, eSMC inferred an egg bank lasting 3–18 generations, consistent with empirical observations (Brendonck and De Meester 2003). A standard SMC approach neglecting egg-banking would have reported a biased $N_{e}$ trajectory, and obscured the role of egg banks in maintaining genetic variation.

The eSMC has also been extended to allow for the joint inference of historical population sizes, recombination rates, and selfing (or germination) rates by allowing these parameters to vary through time (teSMC; Strütt et al. 2023). This enables the dating of transitions from outcrossing to selfing based on temporal changes in the ratio of recombination to mutation rate. Returning to *A. thaliana*, Strütt et al. estimated that a transition to self-fertilization occurred approximately 600 kya. Since teSMC can distinguish historical changes in $N_{e}$ from changes in self-fertilization rates, it also detected a previously-unrecognized population decline coinciding with the transition from outcrossing to selfing. This demonstrates how incorporating time-varying vectors of ecologically-relevant parameters can enhance the resolution of SMC-based inference and our understanding of mating system evolution.

eSMC has also been extended to accommodate systems where a few individuals produce a large proportion of offspring (SM*β*C; Korfmann et al. 2024). This can be caused by skewed offspring distributions—as are seen in plants, invertebrates, prokaryotes, and fish—or strong selection events. SM*β*C employs the *β*-coalescent to allow long range dependencies between coalescent trees along the genome, which are indicative of multiple merger events. SM*β*C has been shown to distinguish between the influences of skewed offspring distribution and positive selection while simultaneously modeling historical population sizes to a high degree of accuracy (Korfmann et al. 2024).

## SMC with Lineage Partitioning

**Fig. 4.**
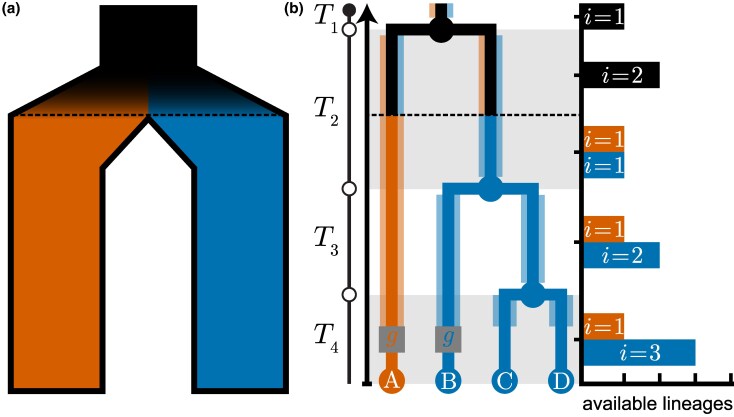
Illustrative example of lineage partitioning. a) Lineages are partitioned into colored classes (vermilion or blue) where cross-color coalescence is restricted until after the barrier (black dashed line) arose. b) Opportunities for coalescence in a lineage partitioning model, conditioned on a recombination event represented by the grey square node *g*, with outcomes stratified by the colored class of *g* (vermilion or blue). Starting from the time of the recombination event, the shading behind each branch indicates the colored class identity of the ancestral lineage available for coalescence. With respect to each colored class, where the floating lineage coalesces depends on the duration of each epoch ($T_{i}$, delineated by alternating backgrounds), and the number of ancestral lineages from the same colored class available during that epoch. The histogram shows the number of ancestral lineages *i* available for coalescence in each epoch stratified by colored class, $T_{i}$, which is proportional to the coalescence rate $i/2N_{e}$.

Under the original SMC model, any lineage may undergo recombination, and that lineage is permitted to re-coalesce with itself or any (older) lineages with overlapping or adjacent ancestral material in the current genomic interval. However, it is possible to define an SMC-like process where lineages are partitioned into different labeled classes—e.g., by carrier status for a chromosomal inversion, or membership of separated populations. In these models, lineages are imagined to have different colors throughout time, where the color is reflective of the lineage’s class membership at any given time. Recombination and/or coalescence events can then be modulated to occur at different rates between lineages of the same or different color. We refer to this type of modification as the SMC with lineage partitioning (Fig. 4).

### Modeling and Detecting Structural Variation with the SMC

Peischl et al. (2013) introduced an SMC variant that uses a lineage partitioning approach to model polymorphic inversions. Each lineage is assigned to a specific color—standard or inverted—and coalescence events are restricted to occur only within a color. While coalescence is not allowed between lineages of different colors, recombination events are not strictly limited to lineages of the same color. Accordingly, the model incorporates both homokaryotypic recombination, where lineages share the same chromosomal arrangement, and heterokaryotypic recombination, in which gene flux (i.e., the movement of genetic material between different chromosomal arrangements; Navarro et al. 1997) is modulated by the location of the event within the inversion (Novitski and Braver 1954). The latter occurs at a much lower rate than the former, but can still occur through mechanisms such as multiple crossovers (Ashburner 1989) or gene conversion (Chovnick 1973). Separating lineages by color reflects the underlying biology of inversions, where recombination is highly suppressed between inversion carriers and noncarriers.

Although this model has only been derived for a single population, and not yet applied to any empirical data, it has generated novel results about LD. Peischl et al. (2013) studied the correlation in coalescence times at pairs of sites across an inverted region generated by their method. They found that, unlike standard models of noninverted regions, where LD decays with the physical distance between sites, an inverted region can maintain long-distance associations between sites. This pattern of LD arises because gene flux events are effectively migration events between the standard and inverted classes of lineages, breaking down the associations between sites. However, unlike migration events, the rate of gene flux events varies spatially along the inversion. Gene flux is more likely to occur in the interior of the inversion, and less likely near the breakpoints. By extending the SMC to model inversions, Peischl et al. (2013) demonstrated that the strength of LD between sites on an inversion depends on both the physical distance between sites and their spatial location on the inversion.

In contrast to modeling the suppression of recombination between inversion carriers and noncarriers, Ignatieva et al. (2025) used the SMC to detect distortions in genealogies resulting from the localized suppression of recombination induced by structural variants (SVs), thereby enabling their identification and analysis in empirical data. The crux of the method (implemented in the software tool DoLoReS) is that, in the case of an inversion, recombination is suppressed in individuals with only one copy of the inverted orientation. If a recombination event occurs on a lineage within the clade of chromosomes carrying the inversion, it is highly likely to coalesce with another lineage within the clade of inverted samples. Similarly, if a recombination event occurs on a branch that does *not* harbor the inversion, it will likely *not* coalesce with any lineages in the clade of inverted samples. Consequently, samples that harbor an inversion should persist as a clade in the ARG for longer stretches of the genome than would otherwise be expected.

To identify such signals of locally suppressed recombination, Ignatieva et al. (2025) used the SMC to derive the distribution of the genomic span for a given clade, conditional on the local tree where the clade first appears. For a given input genealogy, DoLoReS then computes the genomic span of each observed clade, and uses the derived distribution to compute a *p*-value which indicates whether the clade has a longer genomic span than expected.

Using a genealogy reconstructed from human sequencing data (Speidel et al. 2019), DoLoReS identified 50 regions of the genome with strong signals of suppressed recombination. This includes the well-known inversion on 17q21.31, and a known region of complex structural variation on 16p12.2, as well as a novel 760 kb inversion on 10p22.3 which is common in South Asian populations—and which reads-based methods have failed to detect due to lack of strong signal from reads spanning the inversion breakpoints. The method also identified a number of regions with locally suppressed recombination that did not show strong evidence of structural variation; further analysis revealed that the observed signals may arise through expression-dependent suppression of crossovers within genes expressed in meiosis.

### SMC-based Inference with Population Structure

A widely-adopted method, MSMC-IM (Wang et al. 2020) uses a lineage partitioning strategy to model lineages from two populations and infer a time-dependent migration rate estimate between them. Input sequences are colored by which of the two populations they were sampled from. Rather than restricting coalescence between lineages of different colors, as in the case of inversions (Peischl et al. 2013), MSMC-IM expects fewer coalescence events to happen between lineages from different populations, as these events reflect migration. MSMC-IM uses the SMC-based method MSMC2 (Malaspinas et al. 2016; Wang et al. 2020) to infer three separate rates of coalescence: within each population and between the two populations. It then uses these three histories to fit an isolation-migration model (IM; based on Hobolth et al. 2011). The fitting process effectively converts the three coalescence rates inferred from MSMC2 into effective population size histories for each population and the migration rate between them. MSMC-IM has been applied to both intra- and inter-specific questions (Wang et al. 2022; Lescroart et al. 2023; Crossman et al. 2024), leading to novel population genomic and phylogeographic inference. Specifically, researchers have used MSMC-IM to estimate the timing of divergence or secondary contact and to quantify the rate or magnitude of these events, thereby linking demographic history to climatic, ecological, or anthropogenic change. Comparing these parameters among population pairs across a species’ range enables inference of geographic patterns and the spatial scale of evolutionary processes, which can be especially informative when integrated with knowledge of habitat type. Below, we highlight specific use cases.

Mutation rate and generation time are key parameters for SMC interpretation and can substantially affect the magnitude and timing of inferred demographic events (Kovacs et al. 2025). Yet accurate empirical estimates are not always available for nonmodel species. One such species is the mosquito *Aedes aegypti* for which a human specialist *Ae. aegypti aegypti* is a major vector for several viral pathogens. MSMC-IM was used to understand the evolution of the human specialist population from the generalist *Ae. aegypti formosus*; however, the authors first needed to estimate these key parameters. To do this they created MSMC-IM curves from African and South American populations of *Ae. aegypti aegypti*, and calibrated the curve based on the timing of the human slave trade (Rose et al. 2023). Their calibration enabled them to identify that deep population structure in Africa accelerated as the landscape dried and water became associated with human settlements, thereby giving rise to the human specialist. Additional sampling from this system identified the phylogeography of global range expansion of this anthrophilic vector (Crawford et al. 2024).

MSMC-IM is also being applied to study introgression, i.e., the transfer of genetic material between previously isolated populations through hybridization and subsequent back-crossing with one of the parental populations (Dagilis et al. 2022). While unidirectional introgression from polar into brown bears has been previously inferred, the timing was unknown. Analysis of the migration rate curves from MSMC-IM detected the timing of speciation (500 kya–400 kya), and a previously unidentified ancient hybridization affecting global brown bear populations (100 kya; Wang et al. 2022). Similarly, MSMC-IM signatures between wolf and coyote populations identified both speciation and recent hybridization with on-going gene flow that aids in defining evolutionary units for conservation (Vilaça et al. 2023).

The use of MSMC-IM has been limited for questions of selection, but has supported downstream analyses. For example, Puckett et al. (2023) inferred pairwise migration rates among four populations of American black bears to parameterize a set of SLiM (Haller and Messer 2019) models with varying selection coefficients. They found contemporary allele frequencies of the causative locus for brown coat color were best described under a model with a low but positive selection coefficient. Wei et al. (2023) compared the population divergence time due to colonization of the Andes mountains of an endemic tomato species, *Solanum chilense*, to the age of selective sweeps within the population. They found that sweeps in the highlands population were generally closer to the time of colonization of the novel environment than in the age of sweeps in lowland populations.

Shchur et al. (2022) introduced MiSTI as an alternative SMC-based approach to account for population structure, that can infer changes in historical effective population sizes for two populations while correcting for the confounding effects of migration. The method combines coalescence rates inferred from PSMC with the joint site frequency spectrum (jSFS) from two diploid individuals to fit a time-dependent migration model. By incorporating both the PSMC results and the jSFS, MiSTI can disentangle the complex interplay between population size changes and migration, providing estimates of migration-corrected historical population sizes, split time between the populations, and asymmetric migration rates. The crux of MiSTI’s approach lies in its modeling distinctions between different conceptualizations of effective population sizes. Specifically, Shchur et al. (2022) modeled the distinction between the *ordinary* effective population size—i.e., as inferred by methods like PSMC—and the *local* effective population size. The *ordinary* effective population size of an admixed population is comprised of the *local* effective population sizes of the parental populations, which explains why PSMC results can be biased in the presence of population structure (Mazet et al. 2016). In contrast, the *local* effective population size represents the effective population size considering only unadmixed individuals within a parental population—i.e., the inverse coalescence rate conditional on both lineages belonging to the same population at a given time. By modeling the relationship between these two types of effective population sizes as a function of the split time and postseparation migration between the two populations, MiSTI can disentangle the effect of migration on changes in historical population sizes over time.

Rather than inferring demographic histories with migration, Steinrücken et al. (2018) developed diCal-ADMIX, an SMC-based method that conditions on a demographic model to identify genomic regions resulting from introgression. This model-based approach analyzes three populations: a target population (i.e., the proposed recipient of introgression), a putative source population, and a control population presumed to have no genetic contribution from the source population. diCal-ADMIX implements an HMM based on the SMCSD (Paul and Song 2010; Paul et al. 2011; Steinrücken et al. 2013) and requires a prespecified demographic model describing the joint evolutionary history of all three populations. The crux of this method relies on the SMCSD framework: at each locus, haplotypes from the control and source populations form two unchanging “trunk” genealogies, while target population haplotypes are iteratively sampled and absorbed by one of these trunk haplotypes. When iteratively sampling the target haplotypes, lineages belonging to different populations cannot coalesce unless a gene flow event occurs with a given rate at some point in the past. During a gene flow event, the target haplotype is absorbed by one of the two trunk genealogies, where the underlying dynamics of this absorption process are governed by the demographic model. Thus, the trunk genealogy and time of absorption for a target haplotype can change from locus to locus, reflective of the fact that target haplotypes are realized as a mosaic of the source and control populations. diCal-ADMIX models this absorption process for an additional target haplotype, with hidden states representing both the potential absorbing trunk populations and the timing of absorption, and transition and emission probabilities previously defined for the structured SMCSD with migration (Steinrücken et al. 2013). After decoding the HMM, the method marginalizes over absorption times and groups target haplotypes by trunk population, resulting in the probability that each target haplotype derives from either the control population or was introgressed from the source population for each locus. When applied to recover Neanderthal introgressed regions in European and East Asian populations (using African populations as the control), diCal-ADMIX confirmed the well-established pattern of reduced introgressed ancestry on the X chromosome compared to autosomes. Through subsequent gene enrichment analyses and simulations, Steinrücken et al. (2018) demonstrated that this depletion of Neanderthal ancestry is more consistent with the mutational load hypothesis (i.e., higher genetic load in Neanderthals due to smaller effective population size) rather than Dobzhansky–Müller incompatibilities (i.e., invoking reproductive isolation) between archaic and modern humans.

In contrast to the above methods that infer gene flow between given populations, Cousins et al. (2025) introduced cobraa, a method that detects the presence or absence of gene flow from an unsampled population using a single diploid sequence. cobraa is an extension of the PSMC algorithm that explicitly incorporates a model of population structure in the transition matrix of its HMM. This model of population structure assumes the given population was panmictic until time $T_{1}$, at which point a fraction *γ* of lineages instantaneously migrate to an unsampled population. This unsampled population remains isolated until time $T_{2}$, after which all lineages from both populations merge into a single panmictic ancestral population. Using the Baum-Welch algorithm (see Section 1.4), cobraa infers both the historical population size changes for the sampled population, the admixture fraction *γ*, the divergence $T_{2}$ and admixture $T_{1}$ times, and the size of the unsampled population. Importantly, cobraa does not assume *a priori* that the input diploid sequence was sampled from a structured population. Instead, it infers a structured model and an unstructured model (as in PSMC) and compares the log-likelihoods between them to determine which better explains the data. When the structured model provides a better fit, Cousins et al. (2025) introduced a complementary HMM, cobraa-path, which is conceptually similar to cobraa but with hidden states corresponding to both the discrete coalescence time intervals and ancestral lineage path, with modified transition and emission probabilities. The key innovation of cobraa-path is its ability to infer local ancestry states along the genome, identifying regions where none, one, or both lineages traced their ancestry through the unsampled population. In their analysis, Cousins et al. (2025) found that a model where an ancestral population diverges 1.5 Mya and subsequently admixes 300 kya with an unsampled population in a ratio of 80:20% fits human genomic data substantially better than a model without deep admixture. Furthermore, after inferring the genomic regions derived from this admixture event, the authors discovered a negative relationship between this admixed ancestry, the distance to the closest coding sequence, and the high-resolution *B*-map (which quantifies the strength of background selection). They suggest this pattern results from the purging of the unsampled population’s ancestry following the admixture event.

### Using the SMC to Study Speciation

While most SMC-based methods focus on studying the evolutionary history of a single population, several approaches have adapted the SMC framework to study species-level relationships. These methods employ a lineage partitioning strategy defined by the underlying species tree, where lineages are colored by species identity and speciation events create barriers to coalescence.

This strategy was first implemented in CoalHMM (Hobolth et al. 2007; Dutheil et al. 2009), which is parameterized by a three-taxon species tree. Under this model, there are four possible coalescent histories: one lineage sorting, where lineages from the two sister species coalesce (or sort) into their most recent common ancestral species, and three incomplete lineage sorting, which are genealogically discordant with the species tree. CoalHMM models the sequence of local coalescent histories from a three-species alignment, with ancestral states polarized by an additional outgroup species. Unlike standard phylogenetic methods that infer divergence times (i.e., the mean cross-species coalescence time inferred from sequence divergence estimates; Patterson et al. 2006) using genomic data and fossil calibrations (Yang 2007; Ronquist et al. 2012; Bouckaert et al. 2019), CoalHMM directly infers speciation times (i.e., the time of the last gene flow event between the two species’ ancestors; Patterson et al. 2006) and ancestral effective population sizes by leveraging information from local coalescent histories constrained by the species tree. Divergence times inferred from genomic data are typically older than speciation times, as short intervals between speciation events and/or large ancestral effective population sizes can result in coalescent histories of incomplete lineage sorting, where loci from two species coalesce deeper in the past than their actual speciation time (Rivas-González et al. 2023). However, CoalHMM yields biased parameter estimates for the three-species case because coalescent times are modeled as single time points on a given branch (Dutheil et al. 2009).

To address these limitations, Mailund et al. (2011) reparameterized CoalHMM for a two-taxon species tree, where lineages from different species remain restricted from coalescing until the speciation event, but now time is split into discrete bins in an analogous way to PSMC. This two-species model enables more accurate inference of the speciation time and effective ancestral population size (Mailund et al. 2011), and has been extended to incorporate postspeciation migration between species (Mailund et al. 2012).

Building on both the three-species and two-species CoalHMM models, TRAILS jointly infers the topology and coalescent times of local genealogies from a three-species alignment (Rivas-González et al. 2024). Unlike many SMC-based methods that operate on pairs of lineages (or over all possible pairs of lineages), TRAILS infers local genealogies by modeling both the topology (based on the four possible coalescent histories) and the timing of two coalescence events (using discrete time intervals along the species tree). This approach enables unbiased inference of speciation times and ancestral effective population sizes for the three-species tree without requiring fossil calibrations. Moreover, TRAILS can reconstruct the ARG for the three species from its posterior decoding, allowing researchers to not only recover demographic parameters associated with the speciation history, but also study the underlying process of speciation itself.

Diverging from the above methods, which rely on sequence data from two or three species, Cousins et al. (2024b) demonstrated the remarkable applicability of PSMC for studying the speciation history of our own species. By analyzing the posterior distribution of coalescence times from PSMC, the authors showed that a diploid genome sampled from a present day human, chimpanzee, or gorilla does not fully coalesce until beyond 10Mya, and that PSMC is relatively robust to model violations in these ancient time periods. This indicates that coalescent-based inference can be extended much further into the past than previously thought, and in particular that a diploid primate genome contains enough information to elucidate the speciation process with its closest relatives.

## SMC in ARG Inference

The SMC (and its extensions) is a full probabilistic model describing the distribution of sample genealogies. While it is reasonably simple to *simulate* a genealogy under such a model (as described in Section 1, Algorithm 1), the problem of *inferring* or *reconstructing* plausible genealogies conditional on a given sample of sequences is notoriously difficult, due to the huge search space involving both discrete (ARG topology) and continuous (branch length) components. Tremendous progress in ARG inference has been made in the past decade using various simplifying approximations to the SMC (for recent review articles on ARGs, see Brandt et al. 2024; Lewanski et al. 2024; Nielsen et al. 2025; Wong et al. 2024).

Rasmussen et al. (2014) developed ARGweaver, a Bayesian method which outputs a sample from the posterior distribution of possible ARGs for a given genomic sample. ARGweaver relies on the idea of constructing an ARG by “threading” in sequences conditioned on the partial ARG of the previously added sequences, which is conceptually similar to the SMCSD framework (Paul and Song 2010; Paul et al. 2011; Steinrücken et al. 2013). The core threading procedure reconstructs the ARG for *n* lineages by determining the set of branches and coalescence times for the *n*th lineage to join the partial ARG of n−1 lineages, in a manner that is consistent with both the SMC model and the observed genetic variation. Given that the threading procedure is based on the underlying dynamics of the SMC, it is implemented as an HMM, where the hidden states represent the possible branches and coalescence times for the lineage being threaded to join the partial ARG, the transition probabilities describe the probability of branch-specific subtree prune and regraft operations under the SMC, and the emission probabilities correspond to the likelihood of the observed sequence data at the current genomic interval conditioned on the marginal genealogy, which is computed using Felsenstein’s pruning algorithm (Felsenstein 1973, 1981). ARGweaver relies on a Markov chain Monte Carlo (MCMC) sampler to explore the vast space of possible ARGs conditioned on the data and prespecified model parameters (e.g., mutation rate, recombination rate, and the demography). Each MCMC iteration involves removing a branch (or subtree) across all marginal genealogies and then re-threading it using the HMM. This allows for the quantification of uncertainty in both the ARG topology and estimated event times, but the HMM requires time to be discretized, and the procedure is too computationally intensive to scale beyond around 100 haploid genomes (Hubisz and Siepel 2020). To overcome the assumption that all input lineages coalesce in a panmictic ancestral population, ARGweaver-D (Hubisz et al. 2020) was introduced as a demography-aware extension that allows explicit modeling of gene flow events between distinct input lineages. The method was used to infer that ∼3% of the Neanderthal genome derives from an introgression from the early ancestors of modern humans 200 kya–300 kya. More recently, SINGER (Deng et al. 2025a) further enhanced the scalability of the threading approach by improving the efficiency of the HMM and MCMC sampler, enabling ARG inference for up to a couple of hundreds of haploid genomes (with human-like parameters). The inference from ARGweaver has been shown to be powerful for downstream applications such as fitting Bayesian population size history (Palacios et al. 2015) or identifying allele frequency changes to learn about selection (Stern et al. 2019; Vaughn and Nielsen 2024).

Arbores (Heine et al. 2018) is another ARG inference method that relies on the SMC, but adopts an alternative approach to threading: while this has the advantage of not requiring time discretization, it suffers from similar issues with scalability to ARGweaver. ARGinfer (Mahmoudi et al. 2022) reconstructs ARGs under the full CwR model. This is more computationally intensive than ARGweaver, but shows some improvement in the inference of evolutionary parameters (particularly recombination rate).

In contrast to these Bayesian methods that provide posterior samples, other methods which scale to much larger datasets forego the goal of principled uncertainty estimation in the ARG topology and/or event times, and instead rely on approximations to infer a single sensible ARG. For example, Relate (Speidel et al. 2019) and tsinfer/tsdate (Kelleher et al. 2019; Wohns et al. 2022) both use a modified Li and Stephens haplotype copying model (Li and Stephens 2003) to reconstruct local genealogies along the genome. On the other hand, ARG-Needle (Zhang et al. 2023) and Threads (Gunnarsson et al. 2024) reconstruct the ARG using a threading procedure, but instead of using MCMC to sample from the posterior distribution, they use a set of “threading instructions” to sequentially graft a haploid genome into the partial ARG, which results in a single inferred ARG after iteratively threading each haploid genome. While these methods do not provide estimates of uncertainty in both topology and event times like their Bayesian counterparts, they are able to scale to (hundreds of) thousands of haploid genomes. Another challenge is that ARGs inferred by these tools do not contain sufficient information for calculating likelihoods under the SMC, however some recent methodological progress has been made in this direction (Bisschop et al. 2025).

Apart from being key to their methodology, the SMC has also been used as a null model to assess the accuracy of these tools. Quantities calculated from reconstructed ARGs can be compared to their expectations under the SMC. Deng et al. (2021) and McKenzie and Eaton (2025) used the SMC to derive the distribution of the genomic distance between successive local trees, and Ignatieva et al. (2025) derived various distributions characterizing the genomic span of clades and edges in the ARG. This strategy presents an alternative to breaking up reconstructed ARGs into local trees and applying tree-based metrics to measure how close reconstructed ARGs are to the simulated ground truth (Kelleher et al. 2019; Speidel et al. 2019; Brandt et al. 2022).

## Empirical Considerations

Like all inference tools, estimates generated by SMC-based methods are limited by the properties and quality of the available data. This issue is particularly pronounced when studying nonmodel systems with limited existing genomic resources.

### Data Requirements

SMC-based HMMs estimate the coalescence rate (inverse $N_{e}$) at each recombination block from the set of variants observed within each block (see Section 1.4). The number of linked SNPs (HMM observations) in a single recombination block improves the estimate of the genealogy at that locus (HMM hidden state). The total number of recombination blocks across the sequence becomes the number of independent *tMRCA* estimates, and in turn, modulates the precision of the $N_{e}$ inference.

#### Genotyping Resolution

The properties of an organism’s genome and the proportion of its bases that can be confidently genotyped can limit the number of SNPs and blocks available to analyze (Nadachowska-Brzyska et al. 2016). The precision and stability of SMC-derived parameter estimates improve with the ratio of the mutation rate (*θ*) to the recombination rate (*ρ*), scaled by the proportion of the genome that is confidently genotyped (*p*). Although SMC-based methods generally remain robust even with fragmented datasets (Liu and Hansen 2017; Patton et al. 2019) demonstrated that PSMC estimates are typically reliable when θ/ρ>0.5, even when applied to datasets produced by reduced representation sequencing.

Low sequencing coverage can lead to inaccuracies in genotype calls, as factors such as sequencing technology, sample quality, and filtering criteria impact the ability to confidently call genotypes. Population genetic tools such as ANGSD (Korneliussen et al. 2014) have circumvented the need for strict genotype calls by utilizing genotype likelihoods. However, SMC-based methods have yet to adopt a framework that accounts for genotype uncertainty, with ARGweaver and ARGweaver-D being two notable exceptions that can use phred-likelihoods or genotype-likelihoods as input for ARG inference.

A promising way to mitigate issues with sequencing quality is to incorporate heritable markers beyond SNPs. Recently, Sellinger et al. (2024) introduced an SMC-based inference method, SMCtheo, which can in principle accommodate any genomic marker, provided that its occurrence can be modeled as a homogeneous Poisson mutation process along the genome and through time, thereby making it informative of the underlying genealogy. Analyzing hyper-mutable markers alongside SNPs has the potential to improve accuracy of inferred demographic histories over more recent timescales. For instance, SMCtheo has been successfully applied with Single Methylated Polymorphisms (SMPs). Sellinger et al. (2024) used both SNP and SMP markers simultaneously to estimate recent population bottlenecks in *A. thaliana*. However, not all hyper-mutable markers are suitable; for example, the lengths of Differentially Methylated Regions surpass the typical genomic distances between recombination events, making them incompatible with the SMC framework.

#### Genome Phasing

Even with high-quality genotype calls, whether the genomic data is phased or unphased is a key constraint. Because the coalescent process models the ancestral relationships between sampled *haplotypes*, rather than individuals, many SMC-based methods require phased data. This requirement limits researchers, as statistical phasing is only possible for large sample sizes, and its alternatives require large, phased reference panels. Meeting either condition can be challenging for systems outside of humans. A notable exception is PSMC and its variants, which only require homozygous/heterozygous calls from single diploid individual. MSMC2 can infer historical population sizes using unphased genomes, but its inferences are restricted to within-individual comparisons (i.e., not across pairs of genomes). This mode results in lower resolution compared to phased datasets, and cannot be used for population separation analyses or inference of historical migration rates using MSMC-IM.

Nevertheless, there are a handful of SMC-based methods that circumvent the necessity of phased data. One such approach is MiSTI, which provides an attractive alternative to MSMC-IM. MiSTI only considers two diploid individuals, and bypasses the need for phasing by utilizing coalescence rates inferred by PSMC along with the jSFS between the two individuals. Other SMC-based methods capable of handling unphased data include ARGweaver and ARGweaver-D. When the input data is unphased, these methods perform ARG inference by integrating over all possible phasings during their threading procedure.

### Recency Drawback

A drawback of many SMC-based methods is their limited resolution in recent time frames. SMC-based inference works by inferring a local coalescence rate between two sequences from the mutation density (Section 1.4). When considering only two sequences, the majority of coalescence events are expected to occur in the more distant past, resulting in a dearth of information for SMC-based methods to exploit for making accurate inferences in more recent time periods (Nadachowska-Brzyska et al. 2016; Terhorst et al. 2017; Patton et al. 2019; Santiago et al. 2020). This issue is particularly concerning for conservation research, which often relies on a limited number of sequences to study the drivers of recent changes in population sizes.

While this limited resolution in recent time frames is most pronounced in SMC-based methods that make inferences from a single diploid individual (e.g., PSMC and its subsequent variants), methods such as MSMC2 have better resolution for this time frame because they make inferences from multiple individuals. However, MSMC2’s ability to infer recent changes in population sizes is confounded by phasing errors (Terhorst et al. 2017). In response to this confounding effect of phasing errors on demographic inference, Terhorst et al. (2017) developed SMC_++_, as a phase-invariant alternative. This strategy leverages information from the rest of the samples to inform the genealogies of the haplotype pair being analyzed. Specifically, it improves the reliability of $N_{e}$ estimates, especially for notoriously difficult recent time-scales (Terhorst et al. 2017; Patton et al. 2019). SMC_++_ also builds on PSMC by accommodating larger sample sizes and applying regularization to improve estimation error (e.g., fitting smooth splines instead of assuming piecewise constant population sizes).

Outside of the inherent theoretical challenges, SMC-based methods can also produce biased inferences of $N_{e}$ on more recent time scales due to a lack of *a priori* demographic knowledge for the study system, or less than ideal choices of parameter values for the inference method (Parag and Pybus 2019). For example, Hilgers et al. (2025) reported a distinctive pattern of peaks in $N_{e}$ followed by a dramatic decline in recent time scales inferred by PSMC across many studies. Through an extensive simulation-based and empirical sensitivity analysis, Hilgers et al. (2025) found that these biased results could be explained by unobserved population structure or solely relying on PSMC’s default settings for specifying the discrete time windows, which were designed for studying humans. Mitigation strategies include changing the discretization of time to identify statistical artifacts in recent time bins and validating results with simulated data. However, accurately inferring recent demographic changes remains a challenge for all SMC-based approaches that solely rely on a single diploid sequence (Sellinger et al. 2021; Hilgers et al. 2025).

#### SFS as a Summary Statistic for Recent Times

An alternative to SMC-based methods are those that rely on the distribution of allele frequencies in a population, i.e., the site frequency spectrum (SFS). SFS-based approaches are especially useful for recent temporal resolution, as the SFS is a more informative summary statistic for recent demographic events (i.e., within the last 100 generations) (Beichman et al. 2018; Patton et al. 2019; Reid and Pinsky 2022). SFS methods have shown enough resolution to model recent complex migration events in highly heterogeneous human populations (Serradell et al. 2024), and to trace a more complete picture of the demographic context of ancient samples (Kamm et al. 2020; Sümer et al. 2025). However, their accuracy is influenced by the sample size used to construct the SFS (Terhorst et al. 2015; Beichman et al. 2018; Patton et al. 2019; Reid and Pinsky 2022). SFS-based models assume independence among SNPs and do not rely on linkage disequilibrium, making them a flexible alternative when no reference genome is available, for lower-depth whole genome sequencing, and even for reduced representation sequencing data (Beichman et al. 2018; Liu and Fu 2020; Excoffier et al. 2021; Lesturgie et al. 2022). However, the SFS suffers from identifiability and sensitivity issues (Myers et al. 2008; Bhaskar and Song 2014; Terhorst and Song 2015; Baharian and Gravel 2018; Rosen et al. 2018) which can lead to poorly supported inferences of population history (Cousins and Durvasula 2025; Deng et al. 2025b). Alternatively, LD-based methods can outperform both SMC- and SFS-based approaches; however, these methods gain power and accuracy primarily with larger whole-genome sample sizes and require complete reference assemblies (Beichman et al. 2018; Santiago et al. 2020; Reid and Pinsky 2022; Fournier et al. 2023), creating a practical trade-off.

## Conclusions and Further Directions

The SMC has proven to be a powerful and versatile model, enabling researchers to study questions about demographic history, natural selection, mutation and recombination rates, life history traits, structural variation, population structure, gene flow, and speciation. As SMC-based methods tackle more complex inference tasks and accommodate less stringent data requirements, there is a pressing need to validate these methods considering demographic parameters that reflect more realistic and complex biological and ecological scenarios. While tools are often validated using simulations, these may not fully capture the complexities of natural sequences (Wang et al. 2021). Furthermore, as demonstrated by Hilgers et al. (2025), users must also validate their specific implementation choices with simulations to ensure that inferences are consistent with the observed sequence data, allowing for robust interpretation of results. Given that even modest amounts of phasing errors can bias SMC-based inferences (Terhorst et al. 2017), it would be desirable for future methods to remain agnostic to phase-level resolution. This would avoid potential sources of bias and enable the application of SMC-based methods to a wider range of empirical systems where phasing is unavailable or unreliable. However, recent advances in long-read sequencing technologies (van Dijk et al. 2023; Warburton and Sebra 2023) and the generation of complete telomere-to-telomere reference genomes (Naish et al. 2021; Nurk et al. 2022; Chen et al. 2023; Huang et al. 2023; O’donnell et al. 2023; Liu et al. 2024; Yoo et al. 2025) may soon make phasing far less of a concern for SMC-based inference. These exciting developments, together with the rapid increase of resequencing data, highlight the importance of continuing to develop SMC-based methods that are fast, efficient, and scalable. Approaches such as Gamma-SMC demonstrate how methodological innovations can keep pace with ever-growing genomic datasets, and provide a motivating example for future methods development. At the same time, extending SMC-based methods to handle low-coverage data would also be beneficial, broadening their applicability to more nonmodel systems, which often have fewer genomic resources, as well as to the ever-growing datasets of low-coverage ancient genomes (Mallick et al. 2024).

In addition to addressing data-level biases, model-level biases must also continue to be addressed. Current approaches like MSMC-IM, cobraa, and MiSTI highlight that the assumption of panmixia is a common model violation in SMC-based inference. However, these methods model population structure differently, and further exploration of alternative structured models is warranted. In a similar vein, with TRAILS demonstrating the ability to infer the ARG for three species, extending such approaches to incorporate varying ancestral effective population sizes (as in PSMC-based inferences) and models of speciation with gene flow would be valuable, especially as introgression is now increasingly recognized as a frequent process across the eukaryotic tree of life (Dagilis et al. 2022). While SMC-based methods for simulating and studying structural variation have been developed (Peischl et al. 2013; Ignatieva et al. 2025), further work is needed to make these applicable to long-read sequencing datasets, to more fully understand the evolution of SVs of different types.

Another avenue for methodological work is to directly examine the transition matrix of SMC-based HMMs (Sellinger et al. 2021). ARG inference methods offer a way to estimate this transition matrix directly from data, which has been explored as input for SMC-based inference (Strütt et al. 2023). In certain cases, the posterior distribution can be decoded exactly—without the use of a discrete HMM—which avoids biases introduced by the discretization of time (Ki and Terhorst 2024; Schweiger and Durbin 2023). As shown by Cousins et al. (2025), the transition matrix itself can be used to discern between demographic histories of panmixia versus population structure. This approach highlights the potential for using the transition matrix as a summary statistic within an Approximate Bayesian Computation framework to test more complex competing demographic models.

Methods based on the SMC continue to emerge and prove useful for questions beyond historical effective population size. Accommodating alternative targets and nonstandard coalescence processes has made SMC-based inference methods relevant beyond human population genetics. Concurrently, statistical methods for reconstructing ARGs from sequencing data have turned to the SMC to efficiently generate and evaluate genealogies. We hope to bring awareness of new SMC-derived approaches into ecology, conservation, and natural history to support their creative use in empirical studies, as well as highlight their relevance as we move into the ARG era.

## Data Availability

Code to reproduce Fig. 2b has been uploaded to https://gist.github.com/David-Peede/64c53ebe1f1f31d0376d33c7e9fffdd3. Figures 1–4 have been uploaded to https://github.com/David-Peede/PDiagrams/tree/main/smc-review.

## References

[evaf229-B1] Ashburner M . Drosophila: a laboratory manual. Cold Spring Harbor Press; 1989.

[evaf229-B2] Baharian S, Gravel S. On the decidability of population size histories from finite allele frequency spectra. Theor Popul Biol. 2018:120:42–51. 10.1016/j.tpb.2017.12.008. 29305873

[evaf229-B3] Barroso GV, Dutheil JY. The landscape of nucleotide diversity in *Drosophila melanogaster* is shaped by mutation rate variation. Peer Community J. 2023:3:e40. 10.24072/pcjournal.267.

[evaf229-B4] Barroso GV, Puzović N, Dutheil JY. Inference of recombination maps from a single pair of genomes and its application to ancient samples. PLoS Genet. 2019:15:e1008449. 10.1371/journal.pgen.1008449. 31725722 PMC6879166

[evaf229-B5] Beichman AC, Huerta-Sanchez E, Lohmueller KE. Using genomic data to infer historic population dynamics of nonmodel organisms. Annu Rev Ecol Evol Syst. 2018:49:433–456. 10.1146/ecolsys.2018.49.issue-1.

[evaf229-B6] Bhaskar A, Song YS. Descartes’ rule of signs and the identifiability of population demographic models from genomic variation data. Ann Stat. 2014:42:2469–2493. 10.1214/14-AOS1264. 28018011 PMC5175586

[evaf229-B7] Bisschop G, Kelleher J, Ralph P. Likelihoods for a general class of ARGs under the SMC. Genetics. 2025:iyaf103. 10.1093/genetics/iyaf103. 40439129 PMC12774825

[evaf229-B8] Bouckaert R et al BEAST 2.5: an advanced software platform for Bayesian evolutionary analysis. PLoS Comput Biol. 2019:15:e1006650. 10.1371/journal.pcbi.1006650. 30958812 PMC6472827

[evaf229-B9] Brandt DY, Huber CD, Chiang CW, Ortega-Del Vecchyo D. The promise of inferring the past using the ancestral recombination graph. Genome Biol Evol. 2024:16:evae005. 10.1093/gbe/evae005. 38242694 PMC10834162

[evaf229-B10] Brandt DYC, Wei X, Deng Y, Vaughn AH, Nielsen R. Evaluation of methods for estimating coalescence times using ancestral recombination graphs. Genetics. 2022:221:iyac044. 10.1093/genetics/iyac044. 35333304 PMC9071567

[evaf229-B11] Brendonck L, De Meester L. Egg banks in freshwater zooplankton: evolutionary and ecological archives in the sediment. Hydrobiologia. 2003:491:65–84. 10.1023/A:1024454905119.

[evaf229-B12] Charlesworth B . Fundamental concepts in genetics: effective population size and patterns of molecular evolution and variation. Nat Rev Genet. 2009:10:195–205. 10.1038/nrg2526. 19204717

[evaf229-B13] Charlesworth B, Morgan MT, Charlesworth D. The effect of deleterious mutations on neutral molecular variation. Genetics. 1993:134:1289–1303. 10.1093/genetics/134.4.1289. 8375663 PMC1205596

[evaf229-B14] Chen J et al A complete telomere-to-telomere assembly of the maize genome. Nat Genet. 2023:55:1221–1231. 10.1038/s41588-023-01419-6. 37322109 PMC10335936

[evaf229-B15] Chovnick A . Gene conversion and transfer of genetic information within the inverted region of inversion heterozygotes. Genetics. 1973:75:123–131. 10.1093/genetics/75.1.123. 4202769 PMC1212990

[evaf229-B16] Cohen D . Optimizing reproduction in a randomly varying environment. J Theor Biol. 1966:12:119–129. 10.1016/0022-5193(66)90188-3. 6015423

[evaf229-B17] Cousins T, Durvasula A. Insufficient evidence for a severe bottleneck in humans during the early to middle pleistocene transition. Mol Biol Evol. 2025:42:msaf041. 10.1093/molbev/msaf041. 39921621 PMC11848514

[evaf229-B18] Cousins T, Tabin D, Patterson N, Reich D, Durvasula A. 2024a. Accurate inference of population history in the presence of background selection [preprint]. bioRxiv 576291. 10.1101/2024.01.18.576291.

[evaf229-B19] Cousins T, Scally A, Durbin R. A structured coalescent model reveals deep ancestral structure shared by all modern humans. Nat Genet. 2025:57:856–864. 10.1038/s41588-025-02117-1 40102687 PMC11985351

[evaf229-B20] Cousins T, Schweiger R, Durbin R. 2024b. Deep coalescent history of the Hominin lineage [preprint]. bioRxiv 618932. 10.1101/2024.10.17.618932.

[evaf229-B21] Crawford JE et al 1206 genomes reveal origin and movement of Aedes aegypti driving increased dengue risk. Science. 2025:389:eads3732. 10.1126/science.ads3732. PMC12952217 40966355

[evaf229-B22] Crossman CA, Fontaine MC, Frasier TR. A comparison of genomic diversity and demographic history of the North Atlantic and Southwest Atlantic Southern right whales. Mol Ecol. 2024:33:e17099. 10.1111/mec.v33.20. 37577945 PMC13084989

[evaf229-B23] Cui R et al Phased genome assemblies reveal haplotype-specific genetic load in the critically endangered Chinese Bahaba (Teleostei, Sciaenidae). Mol Ecol. 2024:33:e17250. 10.1111/mec.v33.4. 38179694

[evaf229-B24] da Fonseca RR et al Population genomics reveals the underlying structure of the small pelagic European sardine and suggests low connectivity within Macaronesia. Genes (Basel). 2024:15:170. 10.3390/genes15050641. PMC10888339 38397160

[evaf229-B25] Dagilis AJ et al A need for standardized reporting of introgression: insights from studies across eukaryotes. Evol Lett. 2022:6:344–357. 10.1002/evl3.294. 36254258 PMC9554761

[evaf229-B26] Deng Y, Song YS, Nielsen R. The distribution of waiting distances in ancestral recombination graphs. Theor Popul Biol. 2021:141:34–43. 10.1016/j.tpb.2021.06.003. 34186053 PMC8556732

[evaf229-B27] Deng Y, Nielsen R, Song YS. Robust and accurate Bayesian inference of genome-wide genealogies for hundreds of genomes. Nat Genet. 2025a:57:2124–2135. 10.1038/s41588-025-02317-9. PMC12425808 40921789

[evaf229-B28] Deng Y, Nielsen R, Song YS. A previously reported bottleneck in human ancestry 900 kya is likely a statistical artifact. Genetics. 2025b:229:1–3. 10.1093/genetics/iyae192. PMC11708913 39679949

[evaf229-B29] Durbin R, Eddy SR, Krogh A, Mitchison G. Biological sequence analysis: probabilistic models of proteins and nucleic acids. Cambridge University Press; 1998.

[evaf229-B30] Durvasula A et al African genomes illuminate the early history and transition to selfing in Arabidopsis Thaliana. Proc Natl Acad Sci USA. 2017:114:5213–5218. 10.1073/pnas.1616736114. 28473417 PMC5441814

[evaf229-B31] Dutheil JY . On the estimation of genome-average recombination rates. Genetics. 2024:227:iyae051. 10.1093/genetics/iyae051. 38565705 PMC11232287

[evaf229-B32] Dutheil JY, Ganapathy G, Hobolth A, Mailund T, Uyenoyama MK, Schierup MH. Ancestral population genomics: the coalescent hidden Markov model approach. Genetics. 2009:183:259–274. 10.1534/genetics.109.103010. 19581452 PMC2746150

[evaf229-B33] Excoffier L, Marchi N, Marques DA, Matthey-Doret R, Gouy A, Sousa VC. *fastsimcoal2* : demographic inference under complex evolutionary scenarios. Bioinformatics. 2021:37:4882–4885. 10.1093/bioinformatics/btab468. 34164653 PMC8665742

[evaf229-B34] Felsenstein J . Maximum likelihood and minimum-steps methods for estimating evolutionary trees from data on discrete characters. Syst Biol. 1973:22:240–249. 10.1093/sysbio/22.3.240.

[evaf229-B35] Felsenstein J . Evolutionary trees from DNA sequences: a maximum likelihood approach. J Mol Evol. 1981:17:368–376. 10.1007/BF01734359. 7288891

[evaf229-B36] Fisher RA . XXI.—on the dominance ratio. Proc R Soc Edinb. 1923:42:321–341. 10.1017/S0370164600023993.

[evaf229-B37] Fournier R, Tsangalidou Z, Reich D, Palamara PF. Haplotype-based inference of recent effective population size in modern and ancient DNA samples. Nat Commun. 2023:14:7945. 10.1038/s41467-023-43522-6. 38040695 PMC10692198

[evaf229-B38] Griffiths R . The two-locus ancestral graph. Lect Notes Monogr Ser. 1991:18:100–117. 10.1214/lnms/1215459289.

[evaf229-B39] Griffiths R, Marjoram P. Ancestral inference from samples of DNA sequences with recombination. J Comput Biol. 1996:3:479–502. 10.1089/cmb.1996.3.479. 9018600

[evaf229-B40] Griffiths RC, Marjoram P. An ancestral recombination graph. IMA Vol Math Appl. 1997:87:257–270.

[evaf229-B41] Gunnarsson ÁF, Zhu J, Zhang BC, Tsangalidou Z, Allmont A, Palamara PF. 2024. A scalable approach for genome-wide inference of ancestral recombination graphs [preprint]. bioRxiv 610248. 10.1101/2024.08.31.610248.

[evaf229-B42] Haller BC, Messer PW. SLiM 3: forward genetic simulations beyond the Wright–Fisher model. Mol Biol Evol. 2019:36:632–637. 10.1093/molbev/msy228. 30517680 PMC6389312

[evaf229-B43] Harris K, Sheehan S, Kamm JA, Song YS. Decoding coalescent hidden Markov models in linear time. Lect Notes Comput Sci. 2014:8394:100–114. 10.1007/978-3-319-05269-4. PMC4203418 25340178

[evaf229-B44] Hein J, Schierup M, Wiuf C. Gene genealogies, variation and evolution: a primer in coalescent theory. Oxford University Press; 2004.

[evaf229-B45] Heine K, Beskos A, Jasra A, Balding D, De Iorio M. Bridging trees for posterior inference on ancestral recombination graphs. Proc R Soc A. 2018:474:20180568. 10.1098/rspa.2018.0568. 30602937 PMC6304023

[evaf229-B46] Henderson D, Zhu SJ, Cole CB, Lunter G. Demographic inference from multiple whole genomes using a particle filter for continuous Markov jump processes. PLoS One. 2021:16:e0247647. 10.1371/journal.pone.0247647. 33651801 PMC7924771

[evaf229-B47] Hilgers L et al Avoidable false PSMC population size peaks occur across numerous studies. Curr Biol. 2025:35:927–930. 10.1016/j.cub.2024.09.028. 39919744

[evaf229-B48] Hobolth A, Andersen LN, Mailund T. On computing the coalescence time density in an isolation-with-Migration model with few samples. Genetics. 2011:187:1241–1243. 10.1534/genetics.110.124164. 21321131 PMC3070532

[evaf229-B49] Hobolth A, Christensen OF, Mailund T, Schierup MH. Genomic relationships and speciation times of human, chimpanzee, and gorilla inferred from a coalescent hidden Markov model. PLoS Genet. 2007:3:e7. 10.1371/journal.pgen.0030007. 17319744 PMC1802818

[evaf229-B50] Hobolth A, Jensen JL. Markovian approximation to the finite loci coalescent with recombination along multiple sequences. Theor Popul Biol. 2014:98:48–58. 10.1016/j.tpb.2014.01.002. 24486389

[evaf229-B51] Huang Z et al Evolutionary analysis of a complete chicken genome. Proc Natl Acad Sci USA. 2023:120:e2216641120. 10.1073/pnas.2216641120. 36780517 PMC9974502

[evaf229-B52] Hubisz M, Siepel A. Inference of ancestral recombination graphs using ARGweaver. Methods Mol Biol. 2020:2090:231–266. 10.1007/978-1-0716-0199-0. 31975170

[evaf229-B53] Hubisz MJ, Williams AL, Siepel A. Mapping gene flow between ancient hominins through demography-aware inference of the ancestral recombination graph. PLoS Genet. 2020:16:e1008895. 10.1371/journal.pgen.1008895. 32760067 PMC7410169

[evaf229-B54] Hudson RR . Properties of a neutral allele model with intragenic recombination. Theor Popul Biol. 1983:23:183–201. 10.1016/0040-5809(83)90013-8. 6612631

[evaf229-B55] Ignatieva A et al The length of haplotype blocks and signals of structural variation in reconstructed genealogies. Mol Biol Evol. 2025:42:msaf190. 10.1093/molbev/msaf190. PMC12400028 40795034

[evaf229-B56] Kamm J, Terhorst J, Durbin R, Song YS. Efficiently inferring the demographic history of many populations with allele count data. J Am Stat Assoc. 2020:115:1472–1487. 10.1080/01621459.2019.1635482. 33012903 PMC7531012

[evaf229-B57] Kelleher J, Wong Y, Wohns AW, Fadil C, Albers PK, McVean G. Inferring whole-genome histories in large population datasets. Nat Genet. 2019:51:1330–1338. 10.1038/s41588-019-0483-y. 31477934 PMC6726478

[evaf229-B58] Ki C, Terhorst J. Exact Decoding of a Sequentially Markov Coalescent Model in Genetics. J Am Stat Assoc. 2024 119.547:2242–2255. 10.1080/01621459.2023.2252570. 39323740 PMC11421421

[evaf229-B59] Kingman JFC . Mathematics of genetic diversity. Society for Industrial and Applied Mathematics; 1980.

[evaf229-B60] Kingman JFC . On the genealogy of large populations. J Appl Probab. 1982a:19:27–43. 10.2307/3213548.

[evaf229-B61] Kingman JFC . The coalescent. Stoch Process Appl. 1982b:13:235–248. 10.1016/0304-4149(82)90011-4.

[evaf229-B62] Korfmann K, Sellinger TPP, Freund F, Fumagalli M, Tellier A. Simultaneous inference of past demography and selection from the ancestral recombination graph under the beta coalescent. Peer Community J. 2024:4:e33. 10.24072/pcjournal.397.

[evaf229-B63] Korneliussen TS, Albrechtsen A, Nielsen R. ANGSD: analysis of next generation sequencing data. BMC Bioinformatics. 2014:15:356. 10.1186/s12859-014-0356-4. 25420514 PMC4248462

[evaf229-B64] Kovacs TGL, Foley NM, Silver LW, McLennan EA, Murphy WJ, Hogg CJ, Ho SYW. 2025. Mutation rate estimate and population genomic analysis reveals decline of koalas prior to human arrival [preprint]. bioRxiv 654135. 10.1101/2025.05.15.654135. PMC13247528 42262248

[evaf229-B65] Lennon JT, den Hollander F, Wilke-Berenguer M, Blath J. Principles of seed banks and the emergence of complexity from dormancy. Nat Commun. 2021:12:4807. 10.1038/s41467-021-24733-1. 34376641 PMC8355185

[evaf229-B66] Lescroart J et al Extensive phylogenomic discordance and the complex evolutionary history of the neotropical cat genus leopardus. Mol Biol Evol. 2023:40:msad255. 10.1093/molbev/msad255. 37987559 PMC10701098

[evaf229-B67] Lesturgie P, Planes S, Mona S. Coalescence times, life history traits and conservation concerns: an example from four coastal shark species from the indo-pacific. Mol Ecol Resour. 2022:22:554–566. 10.1111/men.v22.2. 34407294

[evaf229-B68] Lewanski AL, Grundler MC, Bradburd GS. The era of the ARG: an introduction to ancestral recombination graphs and their significance in empirical evolutionary genomics. PLoS Genet. 2024:20:e1011110. 10.1371/journal.pgen.1011110. 38236805 PMC10796009

[evaf229-B69] Li H, Durbin R. Inference of human population history from individual whole-genome sequences. Nature. 2011:475:493–496. 10.1038/nature10231. 21753753 PMC3154645

[evaf229-B70] Li N, Stephens M. Modeling linkage disequilibrium and identifying recombination hotspots using single-nucleotide polymorphism data. Genetics. 2003:165:2213–2233. 10.1093/genetics/165.4.2213. 14704198 PMC1462870

[evaf229-B71] Liu J et al The complete telomere-to-telomere sequence of a mouse genome. Science. 2024:386:1141–1146. 10.1126/science.adq8191. 39636971

[evaf229-B72] Liu S, Hansen MM. PSMC (pairwise sequentially Markovian coalescent) analysis of RAD (restriction site associated DNA) sequencing data. Mol Ecol Resour. 2017:17:631–641. 10.1111/men.2017.17.issue-4. 27718335

[evaf229-B73] Liu X, Fu Y-X. Stairway Plot 2: demographic history inference with folded SNP frequency spectra. Genome Biol. 2020:21:280. 10.1186/s13059-020-02196-9. 33203475 PMC7670622

[evaf229-B74] Mahmoudi A, Koskela J, Kelleher J, Chan Y-b., Balding D. Bayesian inference of ancestral recombination graphs. PLoS Comput Biol. 2022:18:e1009960. 10.1371/journal.pcbi.1009960. 35263345 PMC8936483

[evaf229-B75] Mailund T, Dutheil JY, Hobolth A, Lunter G, Schierup MH. Estimating divergence time and ancestral effective population size of Bornean and Sumatran orangutan subspecies using a coalescent hidden Markov model. PLoS Genet. 2011:7:e1001319. 10.1371/journal.pgen.1001319. 21408205 PMC3048369

[evaf229-B76] Mailund T et al A new isolation with migration model along complete genomes infers very different divergence processes among closely related great ape species. PLoS Genet. 2012:8:e1003125. 10.1371/journal.pgen.1003125. 23284294 PMC3527290

[evaf229-B77] Malaspinas A-S et al A genomic history of aboriginal Australia. Nature. 2016:538:207–214. 10.1038/nature18299. 27654914 PMC7617037

[evaf229-B78] Mallick S et al The Allen ancient DNA resource (AADR) a curated compendium of ancient human genomes. Sci Data. 2024:11:182. 10.1038/s41597-024-03031-7. 38341426 PMC10858950

[evaf229-B79] Marjoram P, Wall JD. Fast coalescent simulation. BMC Genet. 2006:7:1–9. 10.1186/1471-2156-7-16. 16539698 PMC1458357

[evaf229-B80] Mazet O, Rodríguez W, Grusea S, Boitard S, Chikhi L. On the importance of being structured: instantaneous coalescence rates and human evolution–lessons for ancestral population size inference? Heredity (Edinb). 2016:116:362–371. 10.1038/hdy.2015.104. 26647653 PMC4806692

[evaf229-B81] McKenzie PF, Eaton DAR. Estimating waiting distances between genealogy changes under a multi-species extension of the sequentially Markov coalescent. Syst Biol. 2025:syaf059. 10.1093/sysbio/syaf059. PMC13016838 40924022

[evaf229-B82] McVean GA, Cardin NJ. Approximating the coalescent with recombination. Philos Trans R Soc Lond B Biol Sci. 2005:360:1387–1393. 10.1098/rstb.2005.1673. 16048782 PMC1569517

[evaf229-B83] Möhle M . Ancestral processes in population genetics: the coalescent. J Theor Biol. 2000:204:629–638. 10.1006/jtbi.2000.2032. 10833361

[evaf229-B84] Myers S, Fefferman C, Patterson N. Can one learn history from the allelic spectrum? Theor Popul Biol. 2008:73:342–348. 10.1016/j.tpb.2008.01.001. 18321552

[evaf229-B85] Nadachowska-Brzyska K, Burri R, Smeds L, Ellegren H. PSMC analysis of effective population sizes in molecular ecology and its application to black-and-white ficedula flycatchers. Mol Ecol. 2016:25:1058–1072. 10.1111/mec.2016.25.issue-5. 26797914 PMC4793928

[evaf229-B86] Naish M et al the genetic and epigenetic landscape of the arabidopsis centromeres. Science. 2021:374:eabi7489. 10.1126/science.abi7489. 34762468 PMC10164409

[evaf229-B87] Navarro A, Betrán E, Barbadilla A, Ruiz A. Recombination and gene flux caused by gene conversion and crossing over in inversion heterokaryotypes. Genetics. 1997:146:695–709. 10.1093/genetics/146.2.695. 9178017 PMC1208008

[evaf229-B88] Nielsen R, Vaughn AH, Deng Y. Inference and applications of ancestral recombination graphs. Nat Rev Genet. 2025:26:47–58. 10.1038/s41576-024-00772-4. 39349760 PMC12036574

[evaf229-B89] Nordborg M . Linkage disequilibrium, gene trees and selfing: an ancestral recombination graph with partial self-fertilization. Genetics. 2000:154:923–929. 10.1093/genetics/154.2.923. 10655241 PMC1460950

[evaf229-B90] Nordborg M, Charlesworth B, Charlesworth D. The effect of recombination on background selection. Genet Res. 1996:67:159–174. 10.1017/S0016672300033619. 8801188

[evaf229-B91] Nouhaud P, Martin SH, Portinha B, Sousa VC, Kulmuni J. Rapid and predictable genome evolution across three hybrid ant populations. PLoS Biol. 2022:20:e3001914. 10.1371/journal.pbio.3001914. 36538502 PMC9767332

[evaf229-B92] Novitski E, Braver G. An analysis of crossing over within a heterozygous inversion in Drosophila melanogaster. Genetics. 1954:39:197. 10.1093/genetics/39.2.197. 17247476 PMC1209645

[evaf229-B93] Nurk S et al The complete sequence of a human genome. Science. 2022:376:44–53. 10.1126/science.abj6987. 35357919 PMC9186530

[evaf229-B94] O’donnell S et al Telomere-to-telomere assemblies of 142 strains characterize the genome structural landscape in Saccharomyces cerevisiae. Nat Genet. 2023:55:1390–1399. 10.1038/s41588-023-01459-y. 37524789 PMC10412453

[evaf229-B95] Palacios JA, Wakeley J, Ramachandran S. Bayesian nonparametric inference of population size changes from sequential genealogies. Genetics. 2015:201:281–304. 10.1534/genetics.115.177980. 26224734 PMC4566269

[evaf229-B96] Palamara PF, Terhorst J, Song YS, Price AL. High-throughput inference of pairwise coalescence times identifies signals of selection and enriched disease heritability. Nat Genet. 2018:50:1311–1317. 10.1038/s41588-018-0177-x. 30104759 PMC6145075

[evaf229-B97] Parag KV, Pybus OG. Robust design for coalescent model inference. Syst Biol. 2019:68:730–743. 10.1093/sysbio/syz008. 30726979

[evaf229-B98] Patterson N, Richter DJ, Gnerre S, Lander ES, Reich D. Genetic evidence for complex speciation of humans and chimpanzees. Nature. 2006:441:1103–1108. 10.1038/nature04789. 16710306

[evaf229-B99] Patton AH, Margres MJ, Stahlke AR, Hendricks S, Lewallen K, Hamede RK. Contemporary demographic reconstruction methods are robust to genome assembly quality: a case study in tasmanian devils. Mol Biol Evol. 2019:36:2906–2921. 10.1093/molbev/msz191. 31424552 PMC6878949

[evaf229-B100] Paul JS, Song YS. A principled approach to deriving approximate conditional sampling distributions in population genetics models with recombination. Genetics. 2010:186:321–338. 10.1534/genetics.110.117986. 20592264 PMC2940296

[evaf229-B101] Paul JS, Steinrücken M, Song YS. An accurate sequentially Markov conditional sampling distribution for the coalescent with recombination. Genetics. 2011:187:1115–1128. 10.1534/genetics.110.125534. 21270390 PMC3070520

[evaf229-B102] Peischl S, Koch E, Guerrero RF, Kirkpatrick M. A sequential coalescent algorithm for chromosomal inversions. Heredity (Edinb). 2013:111:200–209. 10.1038/hdy.2013.38. 23632894 PMC3746820

[evaf229-B103] Puckett EE et al Genetic architecture and evolution of color variation in American black bears. Curr Biol. 2023:33:86–97.e10. 10.1016/j.cub.2022.11.042. 36528024 PMC10039708

[evaf229-B104] Rasmussen MD, Hubisz MJ, Gronau I, Siepel A. Genome-wide inference of ancestral recombination graphs. PLoS Genet. 2014:10:e1004342. 10.1371/journal.pgen.1004342. 24831947 PMC4022496

[evaf229-B105] Reid BN, Pinsky ML. Simulation-based evaluation of methods, data types, and temporal sampling schemes for detecting recent population declines. Integr Comp Biol. 2022:62:1849–1863. 10.1093/icb/icac144. 36104155 PMC9801984

[evaf229-B106] Rivas-González I et al Pervasive incomplete lineage sorting illuminates speciation and selection in primates. Science. 2023:380:eabn4409. 10.1126/science.abn4409. 37262154

[evaf229-B107] Rivas-González I, Schierup MH, Wakeley J, Hobolth A. TRAILS: Tree reconstruction of ancestry using incomplete lineage sorting. PLoS Genet. 2024:20:e1010836. 10.1371/journal.pgen.1010836. 38330138 PMC10880969

[evaf229-B108] Robinson JA et al Genome-wide diversity in the California condor tracks its prehistoric abundance and decline. Curr Biol. 2021:31:2939–2946.e5. 10.1016/j.cub.2021.04.035. 33989525

[evaf229-B109] Ronquist F et al MrBayes 3.2: efficient Bayesian phylogenetic inference and model choice across a large model space. Syst Biol. 2012:61:539–542. 10.1093/sysbio/sys029. 22357727 PMC3329765

[evaf229-B110] Rose NH et al Dating the origin and spread of specialization on human hosts in Aedes aegypti mosquitoes. Elife. 2023:12:e83524. 10.7554/eLife.83524. 36897062 PMC10038657

[evaf229-B111] Rosen Z, Bhaskar A, Roch S, Song YS. Geometry of the sample frequency spectrum and the perils of demographic inference. Genetics. 2018:210:665–682. 10.1534/genetics.118.300733. 30064984 PMC6216588

[evaf229-B112] Santiago E, Novo I, Pardiñas AF, Saura M, Wang J, Caballero A. Recent demographic history inferred by high-resolution analysis of linkage disequilibrium. Mol Biol Evol. 2020:37:3642–3653. 10.1093/molbev/msaa169. 32642779

[evaf229-B113] Schiffels S, Durbin R. Inferring human population size and separation history from multiple genome sequences. Nat Genet. 2014:46:919–925. 10.1038/ng.3015. 24952747 PMC4116295

[evaf229-B114] Schweiger R, Durbin R. Ultrafast genome-wide inference of pairwise coalescence times. Genome Res. 2023:33:1023–1031. 10.1101/gr.277665.123. 37562965 PMC10538485

[evaf229-B115] Schweizer G et al Population genomics of the maize pathogen ustilago maydis: demographic history and role of virulence clusters in adaptation. Genome Biol Evol. 2021:13:evab073. 10.1093/gbe/evab073. 33837781 PMC8120014

[evaf229-B116] Sellinger T, Johannes F, Tellier A. Improved inference of population histories by integrating genomic and epigenomic data. Elife. 2024:12:Rp89470. 10.7554/eLife.89470.4. 39264367 PMC11392530

[evaf229-B117] Sellinger TPP, Abu-Awad D, Tellier A. Limits and convergence properties of the sequentially Markovian coalescent. Mol Ecol Resour. 2021:21:2231–2248. 10.1111/men.v21.7. 33978324

[evaf229-B118] Sellinger TPP, Awad DA, Moest M, Tellier A. Inference of past demography, dormancy and self-fertilization rates from whole genome sequence data. PLoS Genet. 2020:16:e1008698. 10.1371/journal.pgen.1008698. 32251472 PMC7173940

[evaf229-B119] Serradell JM, Lorenzo-Salazar JM, Flores C, Lao O, Comas D. Modelling the demographic history of human North African genomes points to a recent soft split divergence between populations. Genome Biol. 2024:25:201. 10.1186/s13059-024-03341-4. 39080715 PMC11290046

[evaf229-B120] Shchur V, Brandt DYC, Ilina A, Nielsen R. 2022. Estimating population split times and migration rates from historical effective population sizes [preprint]. bioRxiv 496540. 10.1101/2022.06.17.496540.

[evaf229-B121] Sheehan S, Harris K, Song YS. Estimating variable effective population sizes from multiple genomes: a sequentially Markov conditional sampling distribution approach. Genetics. 2013:194:647–662. 10.1534/genetics.112.149096. 23608192 PMC3697970

[evaf229-B122] Speidel L, Forest M, Shi S, Myers SR. A method for genome-wide genealogy estimation for thousands of samples. Nat Genet. 2019:51:1321–1329. 10.1038/s41588-019-0484-x. 31477933 PMC7610517

[evaf229-B123] Steinrücken M, Paul JS, Song YS. A sequentially Markov conditional sampling distribution for structured populations with migration and recombination. Theor Popul Biol. 2013:87:51–61. 10.1016/j.tpb.2012.08.004. 23010245 PMC3532580

[evaf229-B124] Steinrücken M, Spence JP, Kamm JA, Wieczorek E, Song YS. Model-based detection and analysis of introgressed Neanderthal ancestry in modern humans. Mol Ecol. 2018:27:3873–3888. 10.1111/mec.2018.27.issue-19. 29603507 PMC6165692

[evaf229-B125] Steinrücken M, Kamm J, Spence JP, Song YS. Inference of complex population histories using whole-genome sequences from multiple populations. Proc Natl Acad Sci USA. 2019:116:17115–17120. 10.1073/pnas.1905060116. 31387977 PMC6708337

[evaf229-B126] Stern AJ, Wilton PR, Nielsen R. An approximate full-likelihood method for inferring selection and allele frequency trajectories from DNA sequence data. PLoS Genet. 2019:15:e1008384. 10.1371/journal.pgen.1008384. 31518343 PMC6760815

[evaf229-B127] Strütt S, Sellinger T, Glémin S, Tellier A, Laurent S. Joint inference of evolutionary transitions to self-fertilization and demographic history using whole-genome sequences. Elife. 2023:12:e82384. 10.7554/eLife.82384. 37166007 PMC10306369

[evaf229-B128] Sümer AP et al Earliest modern human genomes constrain timing of Neanderthal admixture. Nature. 2025:638:711–717. 10.1038/s41586-024-08420-x. 39667410 PMC11839475

[evaf229-B129] Terhorst J . Accelerated Bayesian inference of population size history from recombining sequence data. 2025; p. 1–8. 10.1038/s41588-025-02323-x PMC12513830 40954248

[evaf229-B130] Terhorst J, Kamm JA, Song YS. Robust and scalable inference of population history from hundreds of unphased whole genomes. Nat Genet. 2017:49:303–309. 10.1038/ng.3748. 28024154 PMC5470542

[evaf229-B131] Terhorst J, Schlötterer C, Song YS. Multi-locus analysis of genomic time series data from experimental evolution. PLoS Genet. 2015:11:e1005069. 10.1371/journal.pgen.1005069. 25849855 PMC4388667

[evaf229-B132] Terhorst J, Song YS. Fundamental limits on the accuracy of demographic inference based on the sample frequency spectrum. Proc Natl Acad Sci USA. 2015:112:7677–7682. 10.1073/pnas.1503717112. 26056264 PMC4485089

[evaf229-B133] Unneberg P et al Ecological genomics in the Northern krill uncovers loci for local adaptation across ocean basins. Nat Commun. 2024:15:6297. 10.1038/s41467-024-50239-7. 39090106 PMC11294593

[evaf229-B134] Upadhya G, Steinrücken M. Robust inference of population size histories from genomic sequencing data. PLoS Comput Biol. 2022:18:e1010419. 10.1371/journal.pcbi.1010419. 36112715 PMC9518926

[evaf229-B135] van Dijk EL et al Genomics in the long-read sequencing era. Trends Genet. 2023:39:649–671. 10.1016/j.tig.2023.04.006. 37230864

[evaf229-B136] Vaughn AH, Nielsen R. Fast and accurate estimation of selection coefficients and allele histories from ancient and modern DNA. Mol Biol Evol. 2024:41:msae156. 10.1093/molbev/msae156. 39078618 PMC11321360

[evaf229-B137] Vilaça ST et al Tracing eastern wolf origins from Whole-Genome data in context of extensive hybridization. Mol Biol Evol. 2023:40:msad055. 10.1093/molbev/msad055. 37046402 PMC10098045

[evaf229-B138] Wakeley J . Coalescent theory: an introduction. W. H. Freeman; 2008.

[evaf229-B139] Wang K, Mathieson I, O’Connell J, Schiffels S. Tracking human population structure through time from whole genome sequences. PLoS Genet. 2020:16:e1008552. 10.1371/journal.pgen.1008552. 32150539 PMC7082067

[evaf229-B140] Wang M-S et al A polar bear paleogenome reveals extensive ancient gene flow from polar bears into brown bears. Nat Ecol Evol. 2022:6:936–944. 10.1038/s41559-022-01753-8. 35711062

[evaf229-B141] Wang Z et al Automatic inference of demographic parameters using generative adversarial networks. Mol Ecol Resour. 2021:21:2689–2705. 10.1111/men.v21.8. 33745225 PMC8596911

[evaf229-B142] Warburton PE, Sebra RP. Long-read DNA sequencing: recent advances and remaining challenges. Annu Rev Genomics Hum Genet. 2023:24:109–132. 10.1146/genom.2023.24.issue-1. 37075062

[evaf229-B143] Wei K, Silva-Arias GA, Tellier A. Selective sweeps linked to the colonization of novel habitats and climatic changes in a wild tomato species. New Phytol. 2023:237:1908–1921. 10.1111/nph.v237.5. 36419182

[evaf229-B144] Wilton PR, Carmi S, Hobolth A. The SMC’ is a highly accurate approximation to the ancestral recombination graph. Genetics. 2015:200:343–355. 10.1534/genetics.114.173898. 25786855 PMC4423375

[evaf229-B145] Wiuf C, Hein J. Recombination as a point process along sequences. Theor Popul Biol. 1999a:55:248–259. 10.1006/tpbi.1998.1403. 10366550

[evaf229-B146] Wiuf C, Hein J. The ancestry of a sample of sequences subject to recombination. Genetics. 1999b:151:1217–1228. 10.1093/genetics/151.3.1217. 10049937 PMC1460527

[evaf229-B147] Wohns AW et al A unified genealogy of modern and ancient genomes. Science. 2022:375:eabi8264. 10.1126/science.abi8264. 35201891 PMC10027547

[evaf229-B148] Wong Y, Ignatieva A, Koskela J, Gorjanc G, Wohns AW, Kelleher J. A general and efficient representation of ancestral recombination graphs. Genetics. 2024:228:iyae100. 10.1093/genetics/iyae100. 39013109 PMC11373519

[evaf229-B149] Wright S . Evolution in Mendelian populations. Genetics. 1931:16:97. 10.1093/genetics/16.2.97. 17246615 PMC1201091

[evaf229-B150] Yang Z . PAML 4: phylogenetic analysis by maximum likelihood. Mol Biol Evol. 2007:24:1586–1591. 10.1093/molbev/msm088. 17483113

[evaf229-B151] Yoo et al Complete sequencing of ape genomes. Nature. 2025:641:401–418. 10.1038/s41586-025-08816-3 40205052 PMC12058530

[evaf229-B152] Zhang BC, Biddanda A, Gunnarsson ÁF, Cooper F, Palamara PF. Biobank-scale inference of ancestral recombination graphs enables genealogical analysis of complex traits. Nat Genet. 2023:55:768–776. 10.1038/s41588-023-01379-x. 37127670 PMC10181934

